# Integrin α PAT-2/CDC-42 Signaling Is Required for Muscle-Mediated Clearance of Apoptotic Cells in *Caenorhabditis elegans*

**DOI:** 10.1371/journal.pgen.1002663

**Published:** 2012-05-17

**Authors:** Hsiao-Han Hsieh, Tsung-Yuan Hsu, Hang-Shiang Jiang, Yi-Chun Wu

**Affiliations:** 1 Institute of Molecular and Cellular Biology, National Taiwan University, Taipei, Taiwan; 2 Center for Systems Biology, National Taiwan University, Taipei, Taiwan; 3 Research Center for Developmental Biology and Regenerative Medicine, National Taiwan University, Taipei, Taiwan; 4 Institute of Atomic and Molecular Sciences, Academia Sinica, Taipei, Taiwan; University of California San Diego, United States of America

## Abstract

Clearance of apoptotic cells by engulfment plays an important role in the homeostasis and development of multicellular organisms. Despite the fact that the recognition of apoptotic cells by engulfment receptors is critical in inducing the engulfment process, the molecular mechanisms are still poorly understood. Here, we characterize a novel cell corpse engulfment pathway mediated by the integrin α subunit PAT-2 in *Caenorhabditis elegans* and show that it specifically functions in muscle-mediated engulfment during embryogenesis. Inactivation of *pat-2* results in a defect in apoptotic cell internalization. The PAT-2 extracellular region binds to the surface of apoptotic cells *in vivo*, and the intracellular region may mediate signaling for engulfment. We identify essential roles of small GTPase CDC-42 and its activator UIG-1, a guanine-nucleotide exchange factor, in PAT-2–mediated cell corpse removal. PAT-2 and CDC-42 both function in muscle cells for apoptotic cell removal and are co-localized in growing muscle pseudopods around apoptotic cells. Our data suggest that PAT-2 functions through UIG-1 for CDC-42 activation, which in turn leads to cytoskeletal rearrangement and apoptotic cell internalization by muscle cells. Moreover, in contrast to PAT-2, the other integrin α subunit INA-1 and the engulfment receptor CED-1, which signal through the conserved signaling molecules CED-5 (DOCK180)/CED-12 (ELMO) or CED-6 (GULP) respectively, preferentially act in epithelial cells to mediate cell corpse removal during mid-embryogenesis. Our results show that different engulfing cells utilize distinct repertoires of receptors for engulfment at the whole organism level.

## Author Summary

When cells undergo apoptosis, their corpses are quickly recognized and phagocytosed by engulfing cells. Although many cell types, such as muscle cells and epithelial cells, possess the ability to remove apoptotic cells, little is known about the receptors and signaling pathways used for apoptotic cell uptake by these “amateur” phagocytes. We show that, in *Caenorhabditis elegans*, integrin PAT-2/PAT-3 functions as an engulfment receptor in muscle cells. The integrin α subunit PAT-2 mediates both the recognition and subsequent phagocytosis of apoptotic cells. PAT-2 signals through UIG-1 for CDC-42 activation, leading to the cytoskeletal reorganization as the engulfing muscle cell extends pseudopods around the apoptotic cell. Furthermore, in contrast to PAT-2, the other integrin α subunit INA-1 and the engulfment receptor CED-1, both of which appear to act upstream of small GTPase CED-10 (RAC1), predominantly function in epithelial cells to mediate cell corpse removal. Therefore, epithelial cells and muscle cells employ different engulfment receptors for apoptotic cell recognition, downstream signaling, and specific GTPase activation during apoptotic cell removal.

## Introduction

During the development of multicellular organisms, cells that are unnecessary, damaged, or harmful undergo programmed cell death (apoptosis) [1]. Apoptotic cells are recognized and subsequently internalized by engulfing cells [2], [3]. Improper engulfment of apoptotic cells has been linked to diseases: too little engulfment may cause inflammation, autoimmune diseases, and cancers [4]–[6], whereas too much engulfment has been implicated in degenerative diseases [7]–[9].

In flies and mammals, engulfment of apoptotic cells is mediated by “professional” phagocytes, such as mobile macrophages and dendritic cells, or by “amateur” phagocytes, such as muscle cells, epithelial cells, and endothelial cells [10]–[14]. Several mammalian receptors involved in apoptotic cell engulfment have been identified and characterized. Receptors such as BAI1 [15], stabilin-1 [16], stabilin-2 [17], TIM-1 [18], TIM-3 [19], TIM-4 [18], integrins [20], [21], and receptor tyrosine kinase Mer [22] bind to the “eat me” signal, externalized phosphatidylserine (PS) [23], [24], on the surface of apoptotic cells either directly [25], [26] or through bridging molecules [27], [28]. BAI1, integrins, and Mer then signal through the conserved DOCK180/ELMO1/RAC GTPase signaling module to promote the internalization of apoptotic cells [15], [29]–[31], whereas stabilin-1 and stabilin-2 do so through the intracellular adaptor GULP [16]. Other “eat me” signal and receptor pairs for engulfment have been reported. For example, lectin receptors bind to altered sugars on apoptotic cells [32], scavenger receptors to oxidized LDL-like moieties [33], and CD14 to ICAM3 [34]. The *in vivo* role of most of these receptors in the clearance of apoptotic cells and the tissues in which they act at the whole organism level have not been defined.

During the development of a *C. elegans* adult hermaphrodite, 1090 somatic cells are generated, 131 of which undergo apoptosis [35]–[37]. The apoptotic cells are removed by their neighboring cells [35], [38]. Cell types such as hypodermal cells (which constitute the external epithelium), pharyngeal muscle cells, and intestinal cells have been shown to function as engulfing cells [37], [38]. Three partially redundant pathways that regulate the engulfment process have been identified. The first pathway is mediated by two cell-surface proteins CED-1 (mammalian homologue MEGF10) and CED-7 (ABCA1) [39], [40]. CED-1 binds to an apoptotic cell through secreted molecule TTR-52 (transthyretin) and transduces the engulfment signal through the adaptor protein CED-6 (GULP) and DYN-1 (dynamin) to promote the engulfment and degradation of apoptotic cells [41]–[43]. The second pathway is regulated by at least three engulfment receptors, phosphatidylserine receptor PSR-1 [44], Frizzled MOM-5 [45], and integrin INA-1/PAT-3 [46], all of which signal through the adaptor protein CED-2 (CRKII) and the bipartite GEF complex CED-5 (DOCK180)/CED-12 (ELMO) for CED-10 (RAC1) GTPase activation [47]–[52]. Phosphoinositide phosphatase MTM-1 (myotubularin) negatively regulates this pathway by inhibiting the recruitment of CED-12 to the plasma membrane [53], [54]. These two engulfment pathways may converge at CED-10 GTPase, which promotes the actin-based cytoskeleton rearrangement required for phagocytosis of apoptotic cells in engulfing cells [55]. CED-10 activity is negatively regulated by GTPase activating protein SRGP-1 during the engulfment process [56]. Compared to these two major pathways, little is known about the third pathway, which is negatively regulated by the cytoskeletal regulator ABL-1 (Abl), which inhibits the engulfment of apoptotic cells by inhibiting ABI-1 (Abl-interacting protein) and acts independently of CED-10 [57].

Integrins are transmembrane αβ heterodimers that make connections to the extracellular matrix and cytoskeleton and activate several signaling pathways required for multiple cellular processes, including cell adhesion, cell migration, and cell survival [58], [59]. *C. elegans* has two integrin α subunits, INA-1 and PAT-2, and a single β subunit, PAT-3 [60]–[62]. Integrin PAT-2/PAT-3 is a component of muscle attachment complexes and is essential for sarcomere assembly [63], [65] and also acts to direct muscle arm extension [66] and distal tip cell migration [67]. We have recently shown that integrin INA-1/PAT-3 functions as an engulfment receptor for apoptotic cells [46]. Intriguingly, the *pat-3* knockout mutant has a stronger defect in cell corpse engulfment than the *ina-1* mutant [46], raising the possibility that *pat-2* may also mediate the removal of apoptotic cells. In this study, we examined and characterized the role of *pat-2* in cell corpse engulfment and showed that it functions in the muscle-mediated internalization of apoptotic cells and acts through a pathway distinct from the previously known pathways.

## Results

### *pat-2* loss-of-function results in an increased number of embryonic cell corpses

*pat-2(st567)* mutants [64] and worms treated with *pat-2* RNAi are embryonic lethal and show a phenotype of paralyzed arrest at the two-fold stage (Pat), as PAT-2 plays an essential role in body wall muscle assembly and function during embryogenesis [63]–[65]. We tested the involvement of *pat-2* in apoptosis by counting the number of apoptotic cells at the comma and 1.5-fold stages, the two stages at which the majority of embryonic apoptosis occurs [37] and *pat-2* mutant embryos are still developing normally, and found that both *pat-2(st567)* and *pat-2(RNAi)* embryos had a Ced (cell death abnormal) phenotype with increased numbers of apoptotic cells (Table 1). The Ced phenotype of the *pat-2(st567)* mutant was rescued by the transgene *P_pat-2_pat-2::gfp*, in which the *pat-2::gfp* translational fusion construct is expressed under the control of the endogenous *pat-2* promoter *P_pat-2_* (Table 2), confirming that the Ced phenotype of the *pat-2(st567)* mutant was specifically caused by *pat-2* loss of function. The *P_pat-2_pat-2::gfp* transgene also rescued the Pat phenotype of the *pat-2(st567)* mutant (Table 3).

**Table 1:** *pat-2* mutants contain more apoptotic cells during mid-embryogenesis than the wild-type.

Genotype	No. of cell corpses^a^		
	Comma	1.5-fold	Pat^b^
wild-type	9.0±1.1	9.9±1.3	nonPat
*pat-2(RNAi)*	12.9±1.7^**^	13.2±1.6^**^	Pat
*pat-2(st567)* c	13.6±1.6^**^	13.6±1.5^**^	Pat
*pat-2(st567)* d	13.3±1.2^**^	13.4±1.7^**^	Pat
*pat-2(st567)* d treated with *pat-2* RNAi	13.0±1.7^**^	13.5±1.2^**^	Pat
*ced-3(n717)*	0.1±0.3	0.0±0.0	nonPat
*pat-2(st567); ced-3(n717)* e	0.0±0.0	0.1±0.2	Pat
*ced-4(n1162)*	0.1±0.3	0.0±0.0	nonPat
*ced-4(n1162); pat-2(RNAi)*	0.1±0.3	0.2±0.4	Pat
*ina-1(RNAi)*	11.1±1.8^**^	11.7±1.4^**^	nonPat
*pat-2(st567)* d *; ina-1(RNAi)*	14.3±1.5^#^	15.9±1.7^##^	Pat
*pat-3(RNAi)*	14.5±2.0^**^	15.7±2.0^**^	Pat
*pat-3(st564)* f	15.0±1.2^**^	14.8±1.2^**^	Pat
*pat-2(st567)* d *; pat-3(RNAi)*	14.2±1.7	15.4±1.9	Pat

a The number of cell corpses in each genotype was scored at the indicated embryonic stage.

b Embryos that showed normal embryogenesis were scored as nonPat and those paralyzed at the 2-fold stage were scored as Pat.

c Homozygous progeny of *unc-79(e1068) pat-2(st567)/dpy-17(e164)* heterozygous mothers.

d Non-transgenic progeny of *unc-79(e1068) pat-2(st567); Ex[P_pat-2_pat-2::gfp]* mothers.

e Non-transgenic progeny of *unc-79(e1068) pat-2(st567); ced-3(n717); Ex[P_pat-2_pat-2::gfp]* mothers.

f Homozygous progeny of *pat-3(st564)/qC1 dpy-19(e1259) glp-1(q339)* heterozygous mothers. *pat-2*, *ina-1*, and *pat-3* single mutants were compared to the wild-type (^**^ *p*<0.001) and double mutants were compared to the corresponding single mutants (^#^ *p*<0.05 and ^##^ *p*<0.001) at each stage. All comparisons were performed using the unpaired t test. The cell corpse numbers of homozygous *pat-2(st567)^d^* embryos treated with *pat-2* RNAi are not distinguishable from those of *pat-2(st567)^d^* or *pat-2*(RNAi) embryos at the same stage. Data are presented as the mean ± standard deviation (SD) for >15 embryos per stage.

**Table 2:** *pat-2* acts in muscle cells to mediate apoptotic cell engulfment.

Genotype	Transgene	No. of cell corpses^a^	
		Comma	1.5-fold
Wild-type	-	9.0±1.1	9.9±1.3
Wild-type	*P_pat-2_pat-2::gfp*	9.3±1.5	10.5±1.4
*pat-2(st567)* b	*-*	13.3±1.2	13.4±1.7
*pat-2(st567)* c	*-*	13.5±1.1	13.4±1.3
*pat-2(st567)*	*P_pat-2_pat-2::gfp*	9.4±1.2^##^	10.0±1.5^##^
*pat-2(st567)*	*P_unc-54_pat-2::gfp*	9.2±1.3^##^	10.2±1.5^##^
*pat-2(st567)* b	*P_ajm-1_pat-2::gfp*	12.7±1.6^**^	13.1±1.7^**^
*pat-2(st567)* c	*P_unc-54_ced-1::gfp*	13.2±0.9^**^	13.6±1.0^**^
*pat-2(st567)* d	*P_unc-54_ina-1::gfp*	11.5±1.0^##**^	11.8±1.2^##**^
*ina-1(gm144)*	*-*	12.2±1.6	12.5±1.9
*ina-1(gm144)*	*P_unc-54_ina-1::gfp*	13.1±0.8^**^	13.7±0.9^**^
*ina-1(gm144)*	*P_ajm-1_ina-1::gfp*	10.2±0.6^##^	10.5±0.7^##^
*ina-1(gm144)*	*P_ajm-1_pat-2::gfp*	11.4±0.5e #**	11.6±0.5e #**
*ced-1(e1735)*	*-*	19.1±1.7	24.1±1.4
*ced-1(e1735)*	*P_ced-1_ced-1::gfp*	9.4±1.3^##^	9.2±1.0^##^
*ced-1(e1735)*	*P_unc-54_ced-1::gfp*	18.2±1.9^**^	24.1±2.3^**^
*ced-1(e1735)*	*P_ajm-1_ced-1::gfp*	9.6±2.0^##^	10.5±2.2^##^
*ced-1(e1735)*	*P_ajm-1_pat-2::gfp*	19.5±1.0^**^	23.8±0.9^**^

a The number of cell corpses in each genotype was scored at the indicated embryonic stage.

b Homozygous progeny of *unc-79(e1068) pat-2(st567); Ex[P_pat-2_pat-2::gfp]* mothers.

c Homozygous progeny of *unc-79(e1068) pat-2(st567); Ex[P_pat-2_pat-2::mcherry]* mothers.

d Homozygous progeny of *unc-79(e1068) pat-2(st567)/dpy-17(e164)* heterozygous mothers.

e The number of cell corpses was scored in the F1 transgenic progeny of the injected worms, n = 10 at each stage. Transgenic embryos were generated and heat-shocked as described in Materials and Methods. Mutants carrying the transgene were compared to the wild-type (*p<0.05 and **p<0.001) or to mutants without the transgene (^#^ *p*<0.05 and ^##^ *p*<0.001) at each stage. Wild-type carrying the transgene were compared to the wild-type (*p<0.05 and **p<0.001). All comparisons were performed using the unpaired t test. Data are presented as the mean ± standard deviation (SD) for >20 (unless noted above) embryos at each stage.

**Table 3:** Effects of mutant *pat-2* transgenes on the Ced and Pat phenotypes.

Genotype	Transgene	No. of cell corpses^a^		
		Comma	1.5-fold	Pat^b^
Wild-type	*-*	9.0±1.1	9.9±1.3	nonPat
Wild-type	*P_pat-2_pat-2Δcyto::gfp*	11.6±1.8^**^	13.2±1.6^**^	nonPat
Wild-type/ heat shock	*P_hsp_pat-2(ex):mcherry*	10.0±1.5^*^	13.3±1.4^**^	nonPat
Wild-type	*P_unc-54_pat-2(ex):mcherry*	11.3±1.2^*^	12.4±1.0^**^	nonPat
*pat-2(st567)* c	*-*	13.4±1.2	13.4±1.7	Pat
*pat-2(st567)*	*P_pat-2_pat-2::gfp*	9.4±1.2^##^	10.0±1.5^##^	nonPat
*pat-2(st567)*	*P_pat-2_pat-2Δcyto::gfp*	13.0±1.4^**^	13.7±1.7^**^	nonPat

a The number of cell corpses in each genotype was scored at the indicated embryonic stage.

b Embryos that showed normal embryogenesis were scored as nonPat, and those paralyzed at the 2-fold stage were scored as Pat.

c Non-transgenic progeny of *unc-79(e1068) pat-2(st567); Ex[P_pat-2_pat-2::gfp]* mothers. Transgenic embryos were generated and heat-shocked as described in Materials and Methods. Mutants carrying the transgene were compared to the wild-type (*p<0.05 and **p<0.001) or to mutants without the transgene (^#^ *p*<0.05 and ^##^ *p*<0.001) at each stage. Wild-type carrying the transgene were compared to the wild-type (*p<0.05 and **p<0.001). All comparisons were performed using the unpaired t test. Data are presented as the mean ± standard deviation (SD) for >20 embryos at each stage.

*ced-3(n717)* and *ced-4(n1162)*, strong mutations in the pro-apoptotic genes *ced-3* and *ced-4* that block almost all programmed cell death [68], suppressed the phenotype of an increased number of cell corpses in *pat-2(st567)* or *pat-2(RNAi)* embryos (Table 1), showing that the extra cell corpses observed in the *pat-2* mutants were generated by programmed cell death. In contrast, the Pat phenotype of the *pat-2(st567)* or *pat-2(RNAi)* mutants was not suppressed by either the *ced-3* or *ced-4* mutation (Table 1). The fact that the Pat and Ced phenotypes can be uncoupled shows they are probably due to the loss of different *pat-2* functions.

### *pat-2* is required for the removal of embryonic cell corpses

**Figure 1:**
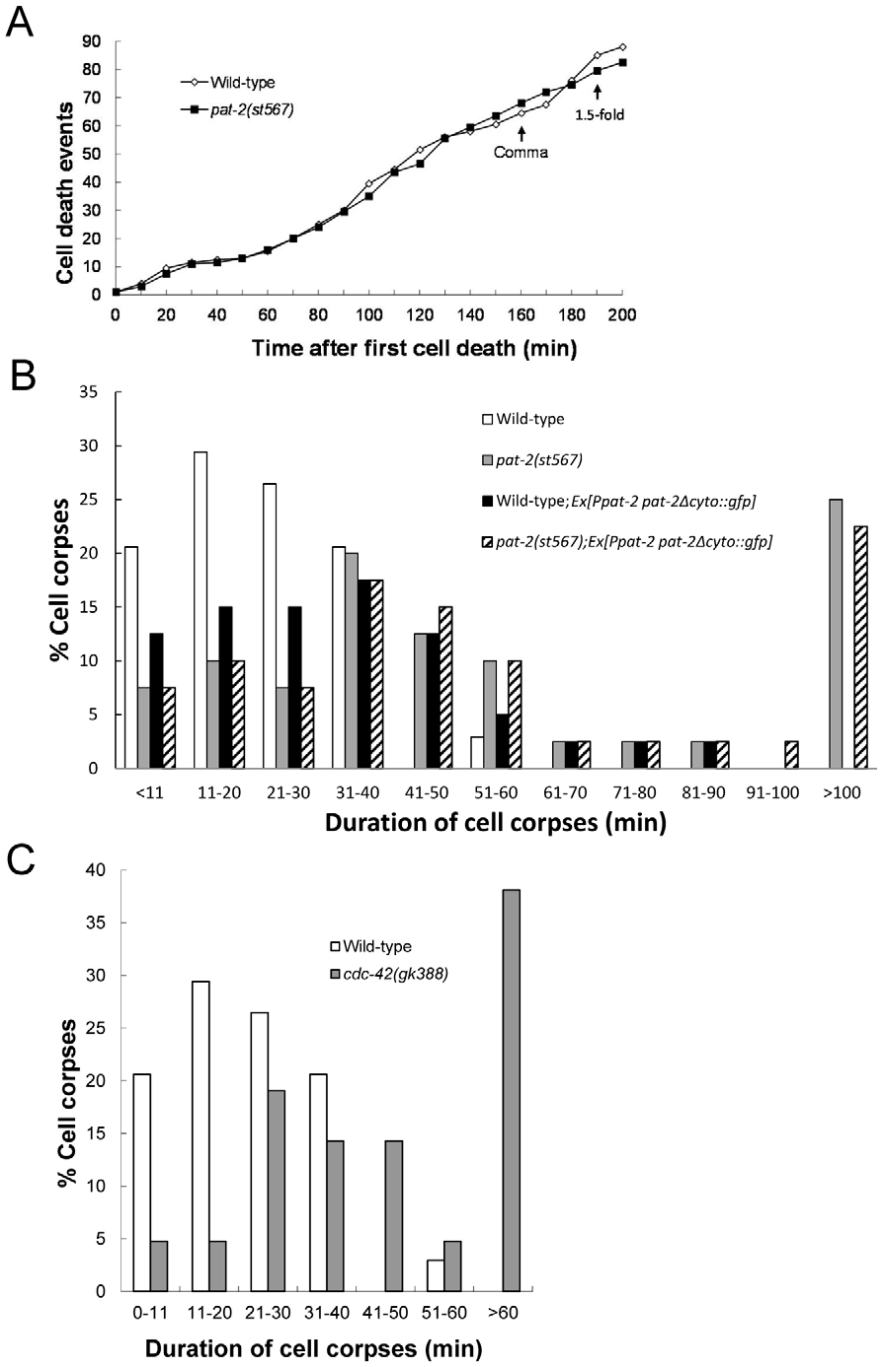
*pat-2* and *cdc-42* mutants are defective in apoptotic cell removal. (A) *pat-2* loss of function does not affect the number of cell death events. Embryonic cell deaths that occurred in the 200 min following the first cell death (up to about the 1.5-fold stage) were followed in wild-type (white rhombi) and *pat-2(st567)* (black squares) embryos. The y axis shows the total number of cell death events at the different time points shown on the x axis. The data shown are the average for two embryos for each genotype. The times corresponding to the comma and 1.5-fold stages are indicated. (B–C) Cell corpses in *pat-2(st567)* (B) and *cdc-42 (gk388)* (C) mutants persist longer than in the wild-type. The persistence of cell corpses that appeared 360–410 min after the first cleavage was recorded using four-dimensional Nomarski microscopy. The y axis shows the percentage of cell corpses that persisted for the time indicated on the x axis. Forty corpses were analyzed for each genotype. The data of wild-type (white bars), *pat-2(st567)* (gray bars); wild-type; *Ex[P_pat-2_pat-2Δcyto::gfp]* (black bars) and *pat-2(st567); Ex[P_pat-2_pat-2Δcyto::gfp]* (slashed bars) are shown in (B) and those of the wild-type (white bars) and *cdc-42(gk388)* (gray bars) in (C).

To determine the cause of the Ced phenotype of the *pat-2* mutant, we performed a time-lapse differential interference contrast (DIC) microscopy analysis of cell corpses in the wild-type and *pat-2(st567)* mutant during embryogenesis prior to the 2-fold stage. We found that, although the timing and number of cell death events were similar in the two types of embryo (Figure 1A), the length of time that the cell corpses persisted was significantly different (Figure 1B). In the wild-type, approximately 97% of cell corpses disappeared within 40 minutes and no cell corpses persisted longer than 60 minutes, whereas, in the *pat-2* embryo, more than 50% of the cell corpses persisted longer than 40 minutes and about 25% longer than 100 minutes. These results demonstrate that *pat-2* functions in the clearance of cell corpses.

### *pat-2* does not function in any previously known pathway for apoptotic cell removal

In addition to PAT-2, *C. elegans* has another integrin α subunit, INA-1, which forms a complex with the single integrin β subunit, PAT-3, on the cell surface [62] and is also required for the clearance of embryonic cell corpses [46]. RNAi inactivation of either *ina-1* or *pat-3* results in increased numbers of apoptotic cells at the comma and 1.5-fold stages [46] (Table 1). The *pat-2(st567)* mutation further increased the number of cell corpses in the *ina-1(RNAi)* mutant, but not in the *pat-3* mutant (Table 1). These results support the notions that PAT-2, like INA-1, acts together with PAT-3 during cell corpse clearance and that the two integrins, PAT-2/PAT-3 and INA-1/PAT-3, function in a partially redundant fashion during the clearance process.

We next determined whether *pat-2* functioned together with previously identified genes to promote cell corpse removal. The two major pathways that regulate cell corpse removal are mediated, respectively, by *ced-1*, *ced-6*, and *ced-7* or *ced-2*, *ced-5*, and *ced-12* [2]. We therefore generated and analyzed double mutants containing either the *pat-2(st567)* or *pat-2(RNAi)* mutation and strong loss-of-function or null alleles of the engulfment *ced* genes for the two pathways. Interestingly, the *pat-2(st567)* or *pat-2(RNAi)* mutation further enhanced the engulfment defect in mutants defective in either pathway (Table 4). This suggests two possibilities. First, *pat-2* could act in both pathways, with the combination of a *pat-2* mutation and a defect in either pathway having an additive effect. Alternatively, *pat-2* could act in a separate pathway that is partially redundant with these two pathways. To distinguish between these two possibilities, we tested whether the *pat-2(st567)* or *pat-2(RNAi)* mutation could increase the engulfment defect in double mutants between the two pathways, such as the *ced-1(e1735); ced-5(n1812)* and *ced-12(tp2); ced-7(n1892)* double mutants. In the first case in which *pat-2* would act in both pathways, we would expect that *pat-2(st567)* or *pat-2(RNAi)* would not enhance the engulfment defect of the double mutants. However, we found that the *pat-2(st567)* or *pat-2(RNAi)* mutation significantly increased the number of cell corpses in both double mutants (Table 4). We therefore conclude that *pat-2* probably functions in a pathway distinct from these two pathways to promote the engulfment of cell corpses.

**Table 4:** *pat-2* promotes apoptotic cell engulfment in a pathway distinct from previously known pathways.

Genotype	No. of cell corpses^a^	
	Comma	1.5-fold
*ced-1(e1735)*	19.1±1.7	24.1±1.4
*ced-1(e1735); pat-2(RNAi)*	23.7±2.3^**^	29.1±4.6^**^
*ced-1(e1735); pat-2(st567)* b	23.0±3.2^**^	28.1±1.8^**^
*ced-6(n1813)*	19.3±2.1	20.3±2.2
*ced-6(n1813); pat-2(RNAi)*	25.3±1.5^**^	25.5±1.8^**^
*ced-7(n1996)*	21.1±1.4	24.2±1.7
*ced-7(n1996); pat-2(RNAi)*	28.8±1.6^**^	30.6±2.0^**^
*ced-2(n1994)*	18.4±2.1	22.8±3.6
*ced-2(n1994); pat-2(RNAi)*	23.1±1.4^**^	25.6±2.4^**^
*ced-5(n1812)*	30.3±2.7	35.3±2.1
*ced-5(n1812); pat-2(RNAi)*	33.1±2.3^**^	42.9±3.3^**^
*pat-2(st567); ced-5(n1812)* c	35.6±3.1^**^	40.6±2.8^**^
*ced-12(n3261)*	21.8±2.4	25.6±2.7
*ced-12(n3261); pat-2(RNAi)*	28.9±1.9^**^	32.1±3.1^**^
*ced-10(n1993)*	23.9±1.6	25.3±2.3
*ced-10(n1993); pat-2(RNAi)*	30.4±1.5^**^	30.6±1.2^**^
*ced-1(e1735); ced-5(n1812)*	33.4±3.9	40.4±2.8
*ced-1(e1735); ced-5(n1812); pat-2(RNAi)*	45.9±2.4^##^	48.4±2.2^##^
*ced-1(e1735); pat-2(st567); ced-5(n1812)* d	41.3±4.6^##^	46.1±5.5^##^
*ced-12(tp2); ced-7(n1892)*	33.2±4.1	40.5±4.5
*ced-12(tp2); ced-7(n1892); pat-2(RNAi)*	44.2±4.5^##^	50.8±1.3^##^
*abi-1(ok640)*	9.3±1.4	10.1±1.1
*abi-1(RNAi)*	10.0±0.9	10.0±0.6
*abi-1(ok640); pat-2(RNAi)*	15.0±1.8^**^	14.5±2.1^*^
*abi-1(ok640); pat-3(RNAi)*	14.8±1.1^**^	14.1±1.2^*^
*abi-1(RNAi); pat-3(st564)* e	17.2±1.3^**^	18.1±1.3^*^
*uig-1(ok884)*	12.1±1.9	13.1±1.4
*cdc-42(gk388)* f	11.9±1.6	13.0±1.1
*uig-1(ok884); pat-2(RNAi)*	12.2±1.5	13.1±0.9
*cdc-42(gk388)* f *; pat-2(RNAi)*	13.0±1.3	13.0±2.4

a The number of cell corpses in each genotype was scored at the indicated embryonic stage.

b Non-transgenic progeny of *ced-1(e1735); unc-79(e1068) pat-2(st567); Ex[P_pat-2_pat-2::gfp]* mothers.

c Non-transgenic progeny of *unc-79(e1068) pat-2(st567); ced-5(n1812); Ex[P_pat-2_pat-2::gfp]* mothers.

d Non-transgenic progeny of *ced-1(e1735); unc-79(e1068) pat-2(st567); ced-5(n1812); Ex[P_pat-2_pat-2::gfp]* mothers.

e Homozygous progeny of *pat-3(st564)/qC1 dpy-19(e1259) glp-1(q339)* heterozygous mothers.

f Homozygous progeny of *cdc-42(gk388)/mIn1[mIs14 dpy-10(e128)]* heterozygous mothers. Double mutants were compared to the corresponding single mutants (*p<0.05 and ** *p*<0.001), and triple mutants were compared to the corresponding double mutants (^##^ *p*<0.001) at each stage. All comparisons were performed using the unpaired t test. Data are presented as the mean ± standard deviation (SD) for >15 embryos per stage.

A recent study has shown that *abi-1* (abl-1 interactor 1) acts in parallel to the two major engulfment pathways during cell corpse removal [57]. Our analysis showed that *pat-2(RNAi)* or *pat-3(st564)* enhanced the engulfment defect in the *abi-1*(null) or *abi-1(RNAi)* embryo (Table 4), showing that *pat-2* and *pat-3* function independently of *abi-1* to mediate the removal of apoptotic cells.

### *pat-2* functions in muscle cells to mediate the removal of apoptotic cells

**Figure 2:**
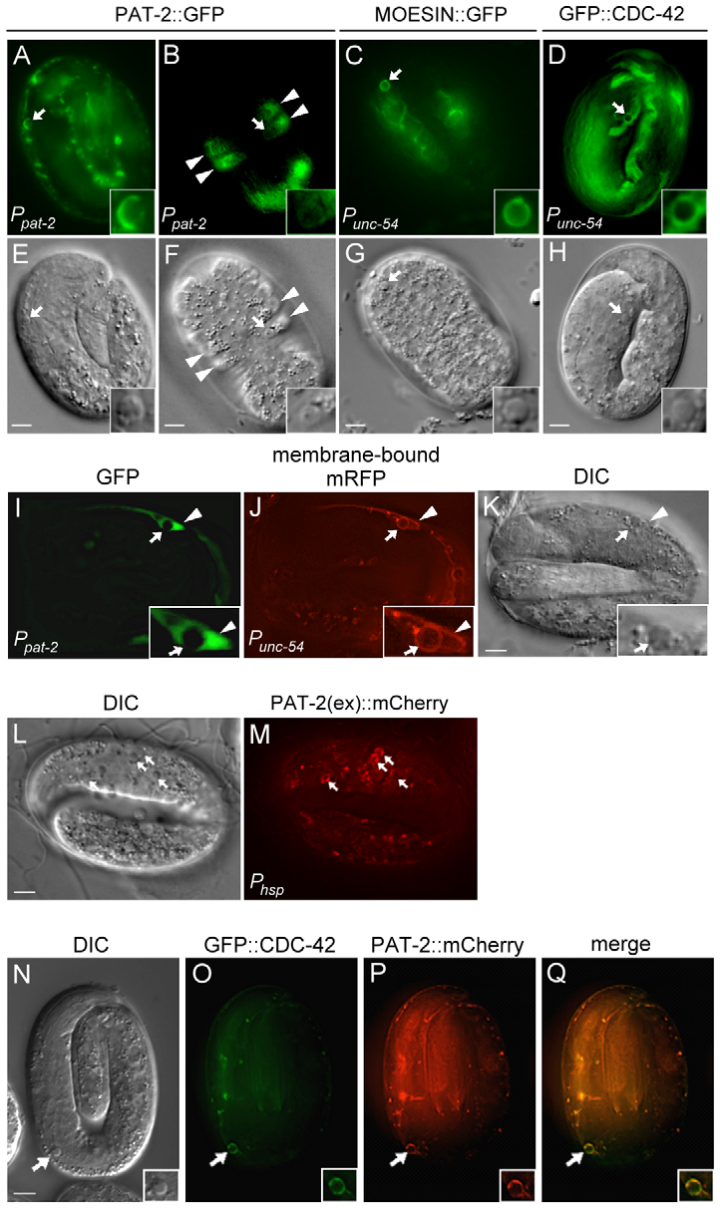
PAT-2 is strongly expressed in muscle cells and clusters around apoptotic cells. (A–H) PAT-2::GFP, MOESIN::GFP, and GFP::CDC-42 are localized to pseudopods around apoptotic cells. GFP (A–D) and DIC (E–H) images of wild-type embryos expressing PAT-2::GFP (A–B), MOESIN::GFP (C), or GFP::CDC-42 (D) under the control of the indicated promoter. Apoptotic cells are indicated by arrows and shown enlarged in the insets. The arrowheads indicate hypodermal cells. (I–K) *P_pat-2_nls::gfp* is not expressed in apoptotic MSpppaaa cells. GFP (I), membrane-bound mRFP (J), and DIC (K) images of an embryo co-expressing *P_unc-54_ced-1::mrfp* and *P_pat-2_nls::gfp*. MSpppaaa cell corpses are indicated by the arrows and shown enlarged in the insets. The arrowheads indicate the nucleus of the engulfing muscle cell. (L–M) The extracellular region of PAT-2 binds to the surface of apoptotic cells. DIC (L) and PAT-2(ex)::mCherry (M) images of a *ced-1(e1735); ced-5(n1812)* double mutant embryo expressing PAT-2(ex)::mCherry under the control of the heat-shock promoter. Apoptotic cells are indicated by arrows. (N–Q) GFP::CDC-42 and PAT-2::mCherry are co-localized to the pseudopods around apoptotic MSpppaaa cells. DIC (N), GFP::CDC-42 (O), PAT-2::mCherry (P), and merged (Q) images of a wild-type embryo co-expressing the transgenes *P_unc-54_ gfp:cdc-42* and *P_pat-2_pat-2::mcherry*. The MSpppaaa cells are indicated by arrows and shown in the insets. All scale bars represent 5 µm.

We next examined the localization of PAT-2 using the *P_pat-2_pat-2::gfp* or *P_pat-2_pat-2::mcherry* transgene, which rescued the Ced and Pat phenotypes of the *pat-2* mutant (Table 3 and data not shown). PAT-2::GFP or PAT-2::mCherry was found to be expressed in body wall muscles and hypodermal cells during embryogenesis (Figure 2A, 2B and Figure S2A). Notably, a strong GFP signal was observed along the surface of apoptotic cells adjacent to muscle cells (Figure 2A and 2E), whereas a relatively weak GFP signal was seen around apoptotic cells engulfed by hypodermal cells (Figure 2B and 2F). Our analysis of embryos expressing the transgene *P_pat-2_nls::gfp*, in which GFP was expressed under the control of *P_pat-2_* and was predominantly localized to the nucleus, indicated that *P_pat-2_* expression was absent from cell corpses during embryogenesis (Figure 2I, 2K, Figure S1 and Text S1). Thus, the PAT-2::GFP signal surrounding apoptotic cells likely originated from the surface of the engulfing cells. The localization of PAT-2::GFP around the surface of apoptotic cells near body wall muscles was not affected by the *ced-1;ced-5* double mutation (Figure S3), consistent with our genetic data showing that *pat-2* acts in parallel to *ced-1* and *ced-5* to mediate cell corpse engulfment. When PAT-2::mCherry and PAT-3::GFP were co-expressed under the control of their respective endogenous promoters, PAT-2::mCherry and PAT-3::GFP were co-localized on apoptotic cells and to the dense bodies (Z-disks) and M-lines of muscle cells (Figure S2A), in agreement with the idea that PAT-2 and PAT-3 form a complex.

In contrast to flies and mammals, *C. elegans* does not have macrophage-like motile phagocytes; instead, apoptotic cells are removed by their neighboring cells [36], [38]. Hypodermal cells, pharyngeal muscle cells, and intestinal cells have been shown to function as engulfing cells [37], [38]. Sinc*e pat-2* was expressed in both hypodermal cells and body wall muscles, we tested whether it functioned in muscle cells and/or hypodermal cells for the removal of apoptotic cells. To this end, we expressed *pat-2* cDNA under the control of the *P_ajm-1_* or *P_unc-54_* promoter and examined the ability of each transgene to rescue the Ced phenotype of the *pat-2* mutant. *P_ajm-1_* is expressed in all epithelia including hypodermal cells [69], whereas *P_unc-54_* is expressed in body wall muscles [70]. We found that expression of *pat-2* by *P_unc-54_*, but not by *P_ajm-1_*, fully rescued the Ced phenotype of the *pat-2(st567)* embryo (Table 2). *P_unc-54_* did not appear to express in apoptotic cells (Figure S4A). These results support the notion that *pat-2* acts in muscle cells to mediate cell corpse removal during embryogenesis.

We next co-expressed PAT-2::mCherry and PAT-2::GFP by the *P_pat-2_* and *P_unc-54_* promoters, respectively, to monitor the PAT-2-mediated and muscle-mediated engulfment processes simultaneously in the 1.5-fold embryos. Approximately 22.7% and 18.1% of the cell corpses were enclosed by the PAT-2::mCherry and PAT-2::GFP circles, respectively, and most of the mCherry and GFP circles were co-localized, suggesting that most, if not all, PAT-2-mediated engulfment involves muscle cells (Table S1).

### CED-1 and INA-1 may function in the hypodermal cell-mediated engulfment of apoptotic cells

CED-1 and INA-1/PAT-3 are engulfment receptors that are expressed in multiple cell types, including hypodermis and body wall muscles [40], [46], but act in pathways in parallel to that involving PAT-2 (Table 1 and Table 4). We therefore tested whether CED-1 and INA-1 acted in specific cell types to mediate the engulfment of apoptotic cells. Expression of *ced-1* in hypodermal cells using the *P_ajm-1_* promoter fully rescued the *ced-1* engulfment defect, whereas expression of *ced-1* in body wall muscles using the *P_unc-54_* promoter did not (Table 2). Similarly, expression of *ina-1* in hypodermal cells, but not muscle cells, rescued the *ina-1* engulfment defect (Table 2). These data suggest that CED-1 and INA-1 preferentially act in hypodermal cells, at least during the comma and 1.5-fold stages. This observation, together with our result that PAT-2 predominantly functions in muscle cells, indicates that different engulfing cells may utilize different engulfment receptors to mediate cell corpse removal.

### Removal of apoptotic MSpppaaa cells by muscle cells requires PAT-2

The mesodermal MSpppaaa cell is generated in the head region about 250 minutes after the first cleavage of a zygote [37]. After circumferential migration to the dorsal midline, the MSpppaaa cell is located near the anterior dorsal muscle cells. Approximately 400 minutes after its generation, the MSpppaaa cell undergoes apoptosis at the four-fold stage [37]. To examine whether the apoptotic MSpppaaa cell was removed by a muscle cell, we expressed membrane-bound monomeric red fluorescent protein (mRFP) in muscle cells using *P_unc-54_*. MSpppaaa cell corpses were found inside the adjacent muscle cells (Figure 2J and 2K), showing that MSpppaaa cell corpses were engulfed by muscle cells. To confirm this result, we tagged PAT-2 with mCherry and GFP and co-expressed PAT-2::mCherry in body wall muscles using *P_unc-54_* and PAT-2::GFP in hypodermal cells using *P_ajm-1_* in wild-type embryos. The PAT-2::mCherry, but not PAT-2::GFP, signal was observed around apoptotic MSpppaaa cells (Figure S5), confirming that apoptotic MSpppaaa cells are engulfed by muscle cells, but not hypodermal cells. The basement membrane between muscle and hypodermis may limit the access of hypodermal cells to the apoptotic MSpppaaa cell for engulfment.

We next examined whether the clearance of apoptotic MSpppaaa cells is defective in *pat-2* embryos by scoring apoptotic MSpppaaa cells in wild-type and *pat-2* embryos at specific embryonic stages. The two stages during which the pharyngeal grinder has just formed and the pharynx is pumping were chosen. The MSpppaaa cell undergoes apoptosis during pharyngeal grinder formation. Pharyngeal pumping begins about 1.5 hours after grinder formation is complete. At the time when pharyngeal grinder formation had just finished, only 19.6% of wild-type embryos contained the MSpppaaa cell corpse and none remained at the time when pharyngeal pumping occurred (Table 5). However, 74.6% of *pat-2* embryos contained the MSpppaaa cell corpse at the time when pharyngeal grinder formation had just finished, and 52.4% still contained a corpse when pharyngeal pumping had started (Table 5). This shows that *pat-2* is important for the removal of apoptotic MSpppaaa cells. In addition, the muscle-specific expression of *pat-2* by *P_unc-54_* significantly reduced the percentage of *pat-2* mutant embryos containing the MSpppaaa cell corpse at the time when pharyngeal grinder formation had just finished or pharyngeal pumping had started (Table 5). In contrast, the hypodermal cell-specific expression of *pat-2* by *P_ajm-1_* failed to do so (Table 5). These results show that *pat-2* functions in muscle cells to mediate the removal of the MSpppaaa cell corpse.

**Table 5:** *pat-2* functions in the muscle-mediated clearance of apoptotic MSpppaaa cells.

Genotype	Transgene	% embryos with the MSpppaaa cell corpse (n)^a^		% MSpppaaa cell corpses with mRFP or GFP circle (n)^b^	
		Grinder formation^c^	Pharyngeal pumping	Grinder formation	Pharyngeal pumping
Wild-type	*-*	19.6 (56)	0 (33)	N.D	N.D
*pat-2(st567)* d	*-*	74.6 (63)	52.4 (84)	N.D	N.D
*pat-2(st567)*	*P_pat-2_pat-2::gfp*	21.4 (42)	0 (17)	N.D	N.D
*pat-2(st567)*	*P_unc-54_pat-2::gfp*	16.7 (48)	0 (10)	N.D	N.D
*pat-2(st567)* d	*P_ajm-1_pat-2::gfp*	76.3 (38)	62.5 (16)	N.D	N.D
Wild-type	*P_unc-54_myri::mrfp*	16.6 (18)	0 (17)	100 (18)	0 (17)
*pat-2(st567)* d	*P_unc-54_myri::mrfp*	76.1 (21)	47.8 (23)	0 (21)	0 (23)
*cdc-42(gk388)* e	*P_unc-54_myri::mrfp*	62.5 (24)	43.7 (16)	0 (24)	0 (16)
*ced-1(e1735)*	*P_unc-54_myri::mrfp*	41.6 (22)	19.0 (21)	100 (20)	0 (21)
Wild-type	*P_pat-2_pat-2Δcyto::gfp*	39.1 (23)	43.3 (30)	66.6 (23)	53.8 (30).
*pat-2(st567)*	*P_pat-2_pat-2Δcyto::gfp*	69.2 (13)	61.5 (13)	0 (13)	0 (13)

a Percentage of embryos with the MSpppaaa cell corpse at the indicated developmental stage, with the number of embryos scored in parentheses.

b Percentage of MSpppaaa cell corpses labeled with the MYRI::mRFP or PAT-2Δcyto::GFPcircle at the indicated developmental stage, with the number of embryos scored in parentheses.

c Embryos at the stage when pharyngeal grinder formation had just finished were scored.

d Homozygous progeny of *unc-79(e1068) pat-2(st567); Ex[P_pat-2_pat-2::gfp]* mothers.

e Homozygous progeny of the heterozygous *cdc-42(gk388)/mIn1[mIs14 dpy-10(e128)]* mothers. The transgenic worms were generated as described in Materials and Methods.

Three cells C1, C2 and C3, called as in reference [42], are generated in the ventral side of embryos at approximately 300–350 min after first cleavage and subsequently undergo apoptosis. Their cell corpses are engulfed by the ventral hypodermal cells ABplaapppp, ABpraapppa and ABplaapppa, respectively [42]. The duration time of these apoptotic cells appeared normal in the *pat-2* mutants (Table S2). This result further supports the notion that *pat-2* preferentially acts in muscle cells but not hypodermal cells for cell-corpse removal. In contrast to *pat-2*, *ced-1* has been previously shown to be important for the hypodermal cell-mediated removal of C1, C2 and C3 cell corpses [42]. In the *ced-1* mutants, the duration time of MSpppaaa cell corpses was slightly longer than that of the wild-types: approximately 19% of MSpppaaa cell corpses still persisted in the *ced-1* mutants during pharyngeal pumping, whereas none remained in the wild-type embryos at this stage (Table 5). Thus, *ced-1* also plays a minor role in the muscle-mediated removal of apoptotic MSpppaaa cells in late embryogenesis.

### Muscle cell-mediated internalization of apoptotic cells is defective in *pat-2* mutants

**Figure 3:**
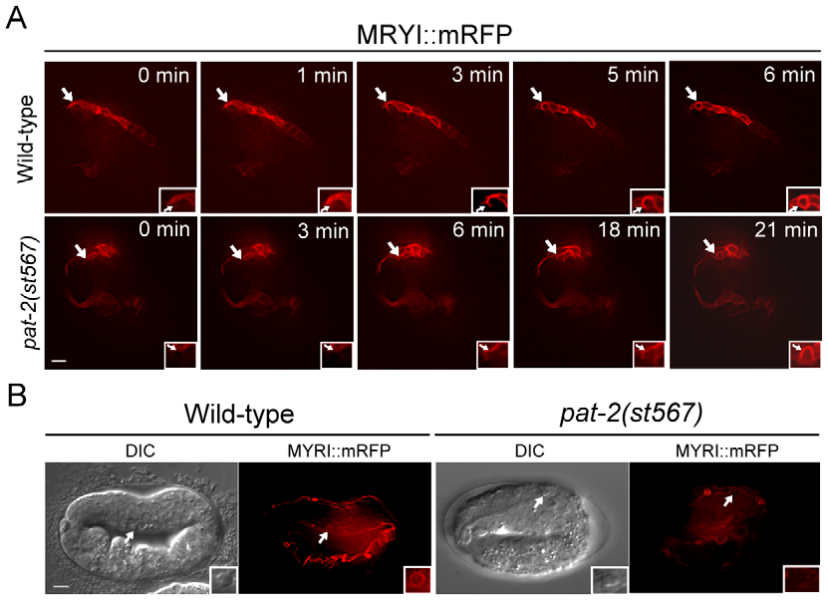
PAT-2 is required for the internalization of apoptotic cells. (A) The formation of MYRI::mRFP circles around cell corpses was followed and time-lapse MYRI::mRFP images of wild-type and *pat-2(st567)* embryos expressing *P_unc-54_myri::mrfp* were shown. The time point immediately prior to the appearance of trace amounts of MYRI::mRFP adjacent to cell corpses was set as 0 min. Apoptotic cells are indicated by arrows and shown enlarged in the insets. (B) The DIC and MYRI::mRFP images of the apoptotic MSpppaaa cells in the wild-type and *pat-2(st567)* embryos expressing *P_unc-54_myri::mrfp*. The MYRI::mRFP circle around the apoptotic MSpppaaa cell was observed in the wild-type but not *pat-2(st567)* embryos. MSpppaaa cell corpses are indicated by arrows and shown in enlarged insets. Both scale bars represent 5 µm.

The persistence of cell corpses in the *pat-2* mutant could be due to a defect in either the internalization or the degradation of the corpse. We used the *P_unc-54_myri::mrfp* translational reporter to express the MYRI::mRFP fusion protein on the surface of muscle cells and followed the membrane processes of a muscle cell around an apoptotic cell using the time-lapse fluorescence microscopy analysis. The comma stage was chosen because the *pat*-*2* embryos at this stage show an increased number of cell corpses and the embryos at this stage do not move around. In wild-type embryos, MYRI::mRFP fusion protein appeared to localize to the growing pseudopods, which eventually formed a circle around an apoptotic cell upon the completion of the internalization process (Figure 3A). The MYRI::mRFP circle formed around an apoptotic cell in approximately 6 minutes. However, in the *pat-2(st567)* mutant embryos, the MYRI::mRFP circle formation took approximately 21 minutes to complete, more than three times longer than that in the wild-type, indicating that the internalization process was compromised.

We further examined the internalization of MSpppaaa cell corpses in the *pat-2* mutant using the *P_unc-54_myri::mrfp* transgene. As shown in Table 5 and Figure 3B, in wild-type embryos, all apoptotic MSpppaaa cells showed the MYRI::mRFP circle at the time when grinder formation had just finished and their cell corpses were cleared at the time of pharyngeal pumping. However, in *pat-2* mutants, no apoptotic MSpppaaa cells had the MYRI::mRFP circle at the time when grinder formation had just finished, and nearly half of MSpppaaa cell corpses still persisted and showed no MYRI::mRFP circle at the time of pharyngeal pumping (Table 5). These data indicate that *pat-2* is required for the internalization of an apoptotic MSpppaaa cell by a muscle cell.

### The extracellular domain of PAT-2 recognizes and binds to apoptotic cells *in vivo*

We then examined whether PAT-2 recognized apoptotic cells to trigger their internalization. To this end, we generated the transgene *P_hsp_pat-2(ex)::mcherry*, in which the coding sequence for the PAT-2 extracellular domain with a signal sequence [PAT-2(ex)] was fused to that of mCherry under the control of the heat-shock promoter *P_hsp_*. The transgene was then introduced into the wild-type and *ced-1(e1735); ced-5(n1812)* double mutant embryos. The *ced-1(e1735); ced-5(n1812)* double mutant embryos contain many persistent apoptotic cells, resulting in a greater chance of seeing PAT-2(ex)::mCherry binding, especially during late embryogenesis when very few cell corpses are present in the wild-type embryos. We found that secreted PAT-2(ex)::mCherry clustered on the surface of apoptotic cells (Figure 2M), albeit with a weaker fluorescence intensity compared to that of PAT-2::GFP (Figure 2A). Approximately 15.2% of apoptotic cells displayed the PAT-2(ex)::mCherry circle in the *ced-1(e1735); ced-5(n1812)* double mutant embryos at the 4-fold stage. A similar percentage (16.6%) was observed in the wild-type embryos at the 1.5-fold stage (Table S1). Thus, PAT-2(ex) recognizes and binds to the surface of apoptotic cells. However, we do not know if binding is direct or indirect.

We further examined whether PAT-2(ex)::mCherry bound to specific apoptotic cells. Approximately 20% of the MSpppaaa cell corpses had a PAT-2(ex)::mCherry circle (Table S1 and Figure S6A), but none of C1, C2 and C3 cell corpses, which are engulfed by hypodermal cells, had a PAT-2(ex)::mCherry circle (Table S1 and Figure S6C and S6D). This result indicates that PAT-2(ex) binds to specific apoptotic cells and may explain why PAT-2 is required for the removal of MSpppaaa (Table 5), but not C1, C2 or C3, cell corpses (Table S2) and why only a subset, but not all, of apoptotic cells are labeled with PAT-2(ex)::mCherry (Figure 2L and 2M). Furthermore, we used the *P_unc-54_* promoter to express PAT-2(ex)::mCherry and observed PAT-2(ex)::mCherry clustering around apoptotic cells including the apoptotic MSpppaaa cell (Figure S4B), despite the fact that the PAT-2(ex)::mCherry signal was not as strong as that expressed by the *P_hsp_* promoter. This result shows that PAT-2(ex)::mCherry expressed in and secreted from muscle cells recognizes, and binds to, the surface of apoptotic cells. Embryos with PAT-2(ex)::mCherry overexpression by either *P_hsp_* or *P_unc-54_* resulted in increased numbers of apoptotic cells at the comma and 1.5-fold stages (Table 3) and delayed the removal of MSpppaaa cell corpses (Figure S6A), indicating that PAT-2(ex)::mCherry overexpression interferes with the normal process of cell corpse engulfment.

The INA-1 extracellular domain, termed INA-1(N) as in reference [46], has been shown to recognize apoptotic cells [46]. To test whether PAT-2 and INA-1 may recognize the same apoptotic cells, we co-expressed PAT-2(ex)::mCherry and INA-1(N)::GFP in embryos using the transgenes *P_hsp_pat-2(ex)::mcherry* and *P_hsp_ ina-1(N)::gfp*. We found that PAT-2(ex)::mCherry was co-localized with INA-1(N)::GFP on some apoptotic cells (Figure S6E). Thus, the extracellular domains of PAT-2 and INA-1 can recognize identical apoptotic cells, although they preferentially function in different cell types for apoptotic cell removal.

### The cytoplasmic domain of PAT-2 is required for cell-corpse engulfment, but not for muscle assembly or contraction

As shown above, PAT-2 binds to apoptotic cells and may serve simply to tether the corpse to an engulfing cell or also initiate a signaling pathway for engulfment. To distinguish between these two possibilities, we tested whether the cytoplasmic domain of PAT-2 was essential for signaling by deleting it and examining the ability of truncated PAT-2Δcyto to rescue *pat-2(st567)* mutants. We generated *pat-2(st567); P_pat-2_pat-2Δcyto::gfp* transgenic animals that expressed the PAT-2Δcyto::GFP fusion protein under the control of the *pat-2* promoter. To our surprise, PAT-2Δcyto::GFP fully rescued the Pat phenotype of the *pat-2(st567)* mutant, but failed to rescue the Ced phenotype (Table 3). The *pat-2(st567); P_pat-2_pat-2Δcyto::gfp* transgenic embryos contained increased numbers of cell corpses (Table 3) at comma and 1.5-fold stages, and some of the cell corpses persisted longer than those of the wild-type embryos (Figure 1B), showing an engulfment defect. The non-Pat phenotype of the transgenic embryos allowed us to count cell corpses at and beyond the 2-fold stage, which is impossible for homozygous *pat-2(st567)* embryos because of developmental arrest. The *pat-2(st567)* embryos carrying the *P_pat-2_ pat-2Δcyto::gfp* transgene also had increased numbers of cell corpses at the 2-, 3- and 4-fold stages (Table S3). Thus, *pat-2* is required for the engulfment of apoptotic cells throughout embryogenesis. In addition, MSpppaaa cell corpses also persisted longer in the *pat-2(st567)*; *P_pat-2_ pat-2Δcyto::gfp* transgenic embryos than those in wild-type embryos. Approximately 61% of the MSpppaaa cell corpses still persisted at the stage of pharyngeal pumping, while none remained in the wild-type embryos at this stage (Table 5).

PAT-2Δcyto::GFP was localized to the surfaces, dense bodies and M-lines of muscle cells in the wild-type and *pat-2* mutants (Figure S7), similar to PAT-2::GFP or PAT-2:: mCherry (Figure S2, S7 and data not shown), suggesting that the cytoplasmic domain is not required for PAT-2 localization. We then used the PAT-2Δcyto::GFP signal to monitor the internalization of MSpppaaa cell corpses by muscle cells. In the wild-type embryos, approximately 66.6% of MSpppaaa cell corpses were enclosed by the PAT-2Δcyto::GFP circle at the time when grinder formation had just finished, whereas no MSpppaaa cell corpses were enclosed by the PAT-2Δcyto::GFP circle in the *pat-2(st567)* embryos at this stage (Table 5 and Figure S8). Thus, the internalization of MSpppaaa cell corpses is defective in the *pat-2(st567); P_pat-2_pat-2Δcyto::gfp* embryos. This result and the aforementioned data together indicate that the PAT-2 cytoplasmic domain is required for the muscle-mediated engulfment of apoptotic cells, but is dispensable for its subcellular localization in muscle cells and function during muscle development.

Expression of the PAT-2Δcyto::GFP fusion protein in the wild-type embryos resulted in increased numbers of cell corpses at the comma and 1.5-fold stages comparable to those seen in *pat-2* mutants, but failed to induce the Pat phenotype (Table 3). In addition, PAT-2Δcyto::GFP also prolonged the duration time of the MSpppaaa cell corpses and interfered with the internalization of the MSpppaaa cell corpses (Table 5). For example, in the wild-type embryos expressing PAT-2Δcyto::GFP, 39% of the MSpppaaa cell corpses persisted at the time when grinder formation had just finished, and 66.6% of these cell corpses were internalized by engulfing muscle cells. However, in the control wild-type embryos, only 16.6% of the MSpppaaa cell corpses were present at this stage, and all the remaining cell corpses were internalized by engulfing muscle cells (Table 5). This result reinforces the specific function of the PAT-2 cytoplasmic domain in the engulfment of apoptotic cells. PAT-2Δcyto::GFP may compete with the endogenous PAT-2 for binding of PAT-3 or apoptotic cells, but fail to initiate signaling for the internalization of cell corpses.

### PAT-2 functions through UIG-1 and CDC-42 to promote the engulfment process

During the engulfment of apoptotic cells, cytoskeletal rearrangement occurs as an engulfing cell extends pseudopods around an apoptotic cell [71]. Rho-family GTPases are important regulators of the actin cytoskeleton [72]. Although CED-10 (RAC1) GTPase is required for cell corpse engulfment [55], [72], our data showing that a *ced-10* mutation enhanced the engulfment defect in the *pat-2(RNAi)* mutant (Table 4) and that *ced-10* overexpression failed to rescue the engulfment defect of the *pat-2(RNAi)* mutant (Table 6) suggest that *pat-2* may act independently of *ced-10* in promoting the cytoskeletal rearrangement required for the internalization process.

**Table 6:** *cdc-42* acts downstream of *pat-2* during cell-corpse engulfment.

Genotype	Transgene	Heat-shock	No. of cell corpses^a^	
			Comma	1.5-fold
Wild-type	*-*	−	9.0±1.1	9.9±1.3
Wild-type	*P_unc-54_gfp::cdc-42*	−	9.3±2.5	9.6±1.2
*cdc-42(gk388)* b	*P_unc-54_gfp::cdc-42*	−	9.2±1.5^**^	9.3±1.1^**^
*cdc-42(gk388)* b	*P_ajm-1_gfp::cdc-42*	−	12.4±0.9	13.3±1.2
*cdc-42(gk388)* b	*P_unc-54_ina-1::gfp*	−	12.0±1.3	13.6±0.5
*pat-2(RNAi)*	*-*	−	12.9±1.7	13.2±1.6
*pat-2(RNAi)*	*-*	+	13.3±1.5	13.2±2.0
*pat-2(RNAi)*	*P_unc-54_gfp::cdc-42*	−	9.3±1.6^**^	10.1±1.4^**^
*pat-2(RNAi)*	*P_hsp_gfp::cdc-42*	+	9.5±1.1^**^	10.6±1.7^**^
*pat-2(RNAi)*	*P_hsp_ced-10*	+	13.2±1.7	13.4±1.6
*pat-2(st567)* c	*P_hsp_ced-10V12*	+	13.9±1.5	13.8±1.3
*ina-1(gm144)*	*-*	−	12.2±1.6	12.5±1.9
*ina-1(gm144)*	*P_ajm-1_gfp::cdc-42*	−	12.3±1.0^d^	12.4±0.9^d^
*ced-10(n3246)*	*-*	−	23.7±2.0	29.8±2.0
*ced-10(n3246)*	*-*	+	23.6±2.4	29.6±3.3
*ced-10(n3246)*	*P_hsp_gfp::cdc-42*	+	23.9±2.2	29.8±2.4
*ced-10(tm597)*	*-*	−	30.2±3.1	38.4±3.6
*ced-10(tm597)*	*P_hsp_ced-10V12*	+	11.5±2.7^**^	12.1±2.2^**^
*ced-1(e1735)*		−	19.1±1.7	24.1±1.4
*ced-1(e1735)*	*P_hsp_gfp::cdc-42*	+	18.5±1.1	23.8±1.9

a The number of cell corpses in each genotype was scored at the indicated embryonic stage.

b Homozygous progeny of the heterozygous *cdc-42(gk388)/mIn1[mIs14 dpy-10(e128)]* mothers.

c Homozygous progeny of the heterozygous *unc-79(e1068) pat-2(st567)/dpy-17(e164)* mothers.

d The number of cell corpses was scored in the F1 transgenic progeny of the injected worms, n = 10 at each stage. Mutants carrying the transgene were compared to mutants without the transgene (*p<0.05 and ** *p*<0.001). The transgenic worms were generated as described in Materials and Methods. Statistical analysis was performed using the unpaired t test. The data are presented as the mean ± standard deviation (SD), n>15 (unless noted above).

We then examined whether actin filament assembly occurred during the muscle-mediated internalization of apoptotic cells. MOESIN has been used in *Drosophila* and *C. elegans* to specifically mark the filamentous form of actin [73], [74]. Analysis of embryos expressing *P_unc-54_moesin::gfp* in which MOESIN::GFP was expressed in muscle cells using *P_unc-54_* showed a MOESIN::GFP circle around apoptotic cells (Figure 2C), demonstrating that filamentous actin assembly occurs as an engulfing muscle cell extends pseudopods along the surface of an apoptotic cell.

A previous study showed that the Rho-family GTPase CDC-42 and UIG-1, a guanine nucleotide exchange factor (GEF) specific for CDC-42, function downstream of PAT-2/PAT-3 signaling for muscle assembly [75]. We next tested the involvement of *uig-1* and *cdc-42* in PAT-2-mediated cell corpse engulfment. The *cdc-42(gk388)* allele has a deletion that eliminates part of the 5′ regulatory sequence, the entire first exon, and part of the first intron of the *cdc-42* gene (*C. elegans* Gene Knockout Consortium), while the *uig-1(ok884)* mutation deletes the region coding for the DH/PH domain, which has the GEF activity [76]. Both alleles are likely to be null. We found that *uig-1* or *cdc-42* embryos contained increased numbers of cell corpses at the comma and 1.5-fold stages (Table 4), similar to those observed in the *pat-2* mutants. A four-dimensional DIC analysis of cell corpse persistence in the *cdc-42(gk388)* embryos revealed a cell-corpse engulfment defect. In the wild-type, 97% of cell corpses disappeared within 40 minutes and none lasted longer than 60 minutes (Figure 1C). However, in the *cdc-42(gk388)* mutants, only 40% of the cell corpses disappeared within 40 minutes and almost 40% persisted longer than 60 minutes (Figure 1C).

We next tested whether *cdc-42* acted in the same pathway as *pat-2* to promote apoptotic cell engulfment. We found that neither *cdc-42* nor *uig-1* increased the number of cell corpses in *pat-2* mutants at the comma and 1.5-fold stages (Table 4), suggesting that *cdc-42* and *uig-1* both act in the same genetic pathway as *pat-2*. Like PAT-2::GFP, the GFP::CDC-42 fusion protein expressed using the transgene *P_unc-54_gfp::cdc-42* was localized in muscle pseudopods around apoptotic cells (Figure 2D). In addition, the transgene *P_unc-54_gfp::cdc-42* rescued the engulfment defect of the *cdc-42(gk388)* mutants, whereas *P_ajm-1_gfp::cdc-42*, which expressed GFP::CDC-42 in hypodermal cells, did not (Table 6). This result suggests that *cdc-42* acts in muscle cells to mediate the engulfment of apoptotic cells. This muscle-specific function of *cdc-42* is further supported by the observation that loss of *cdc-42* results in a defect in the engulfment of apoptotic MSpppaaa cells (Table 5), but not C1, C2 or C3 cells (Table S2). When *P_unc-54_gfp::cdc-42* and *P_pat-2_pat-2::mcherry* were co-expressed, GFP::CDC-42 was co-localized with PAT-2::mCherry along the surface of apoptotic MSpppaaa cells (Figure 2N–2Q). Furthermore, the *P_unc-54_gfp::cdc-42* transgene rescued the engulfment defect of the *pat-2(RNAi)* embryos (Table 6). These results support the model that *cdc-42* functions downstream of *pat-2* in the muscle-mediated engulfment of apoptotic cells.

### CED-10 and CDC-42 are not exchangeable in function during cell-corpse engulfment

Our genetic data suggested that *cdc-42* acts with *pat-2* in the same pathway, whereas *ced-10* functions in a partially redundant manner with *pat-2* to mediate cell corpse engulfment (Table 4). We then tested whether the functions of *ced-10* and *cdc-42* in engulfment were exchangeable when ubiquitously overexpressed. We found that overexpression of *ced-10* or constitutively active *ced-10 V12* by the heat-shock promoter *P_hsp_*, which rescued the *ced-10(n1993)* or *ced-10(tm597)* mutant ([50], Table 6), failed to rescue the engulfment defect of the *pat-2(RNAi)* embryos (Table 6). Reciprocally, overexpression of *cdc-42* by *P_hsp_*, which rescued the *pat-2* mutant (Table 6), did not rescue the engulfment defect of the *ced-10(n3246)* mutants (Table 6). Moreover, overexpression of *cdc-42* by the *P_hsp_* or *P_ajm-1_* promoter also failed to rescue the defective engulfment of the *ced-1(e1735)* or *ina-1(gm144)* mutants, respectively (Table 6). These data suggest that the mechanisms by which *cdc-42* and *ced-10* mediate the actin-based cytoskeletal rearrangement during cell-corpse engulfment are distinct and that their functions are not exchangeable.

### INA-1 and PAT-2 can partially substitute for each other in cell-corpse engulfment

As shown in Figure S6E, the INA-1 and PAT-2 extracellular domains can recognize the same apoptotic cells. We found that overexpression of *pat-2* by the *P_ajm-1_* promoter in hypodermal cells partially rescued the defective engulfment of the *ina-1* mutants and, reciprocally, overexpression of *ina-1* by the *P_unc-54_* promoter in muscle cells also partially rescued the engulfment defect of the *pat-2* mutants (Table 2). This result indicates that *ina-1* and *pat-2* can partially substitute for each other in cell corpse engulfment. Interestingly, overexpression of *ina-1* by the *P_unc-54_* promoter, which partially rescued the engulfment defect of the *pat-2* mutants, did not rescue that of the *cdc-42(gk388)* mutants (Table 6) suggests that *cdc-42* may be necessary for *ina-1* overexpression-induced engulfment in muscle cells. If so, *ina-1* overexpression in muscle cells may promiscuously activate *cdc-42* signaling, which, in turn, leads to the engulfment of apoptotic cells. Nevertheless, *ina-1* overexpression does not efficiently activate the phagocytosis machinery for cell corpse removal in muscle cells since only partial rescue of the *pat-2* engulfment defect by *ina-1* overexpression was observed. Similarly, overexpression of *pat-2* dose not efficiently induce the phagocytosis machinery in hypodermal cells. This may, in part, explain why *pat-2* and *ina-1* preferentially function in different cell types for cell corpse removal, despite that they are expressed in both cell types.

In contrast, overexpression of *ced-1* under the control of the *P_unc-54_* promoter failed to rescue the defective engulfment of the *pat-2* mutants (Table 2). In a reciprocal experiment, overexpression of *pat-2* by the *P_ajm-1_* promoter also failed to rescue the defective engulfment of the *ced-1* mutants (Table 2). These results indicate that *ced-1* and *pat-2* functions are distinct and not exchangeable in cell corpse engulfment.

## Discussion

The engulfment of apoptotic cells requires the recognition and subsequent internalization of apoptotic cells by the engulfing cells. Here, we showed that, in *C. elegans*, the integrin α subunit PAT-2 functions in both the recognition and internalization steps. *pat-2* loss of function resulted in an increased number of embryonic cell corpses due to a defect in cell corpse removal. Our data showed that PAT-2 bound to apoptotic cells through its extracellular domain and initiated downstream signaling via its cytoplasmic domain. We characterized the *pat-2* signaling pathway and identified a previously unassigned function of *cdc-42* and *uig-1* in cell corpse engulfment. We further showed that PAT-2 predominantly functioned in muscle cells to mediate the engulfment process. We propose that binding of PAT-2 to an apoptotic cell results in the recruitment of UIG-1 and the subsequent activation of CDC-42 GTPase, which, in turn, regulates cytoskeletal rearrangement as a muscle cell extends pseudopods around an apoptotic cell.

The finding that truncated PAT-2 lacking the cytoplasmic domain (PAT-2Δcyto::GFP ) fully rescued the Pat phenotype of the *pat-2* mutants, but failed to rescue the engulfment defect (Table 3) argues against the possibility that the engulfment defect of the *pat-2* mutant is a secondary effect caused by abnormality of muscle assembly or organization during embryogenesis. PAT-2 is co-localized with PAT-3 to the dense bodies and M-lines (Figure S2A), which are platforms serving as anchoring sites for signaling/adapter proteins in muscle attachment and organization [65]. Because the deletion of the cytoplasmic domain of PAT-2 affects neither its localization pattern (Figure S7) nor its function in muscle development (Table 3), muscle development probably requires only the transmembrane and extracellular domains, which are likely sufficient for the interaction of PAT-2 with PAT-3 and/or the extracellular matrix. Thus, the PAT-2/PAT-3 integrin likely mediates the intracellular signaling and/or adaptor protein binding through the cytoplasmic domain of PAT-3, but not that of PAT-2, for muscle development. In contrast, the requirement of the cytoplasmic domain of PAT-2 for cell-corpse removal suggests that this domain is important for signaling and/or serves as an anchorage site for adapter proteins during cell-corpse internalization.

The *pat-2(st567)* mutants show a weak engulfment phenotype compared with that of the *ced-1* mutants during embryogenesis (Table 2). One possible explanation for the weak engulfment defect is that *pat-2* predominantly functions in muscle cells, while only a small fraction of cell corpses (e.g. ∼20% of cell corpses in the 1.5-fold stage embryos, as shown in Table S1) are removed by muscle cells. Secondly, other engulfment receptor(s) may act redundantly with *pat-2* in muscle-mediated engulfment. For instance, both PAT-2 and CED-1 are involved in the muscle-mediated internalization of MSpppaaa cells, despite that PAT-2 plays a bigger role than CED-1 (Table 5).

The PAT-2 extracellular region bound to apoptotic cells when PAT-2(ex) was fused to mCherry and overexpressed using the heat shock promoter (Figure 2M). Because exposed PS is detected on the surface of apoptotic MSpppaaa cells (Figure S1B), which are then removed by the PAT-2-mediated engulfment process (Table 5), PAT-2 might recognize exposed PS on apoptotic MSpppaaa cells. Mammalian integrins α_v_β_3_ and α_v_β_5_ have been shown to bind to apoptotic cells via the secreted bridging molecule MFG-E8 [25], [48]. In addition, integrin α_v_β_3_ binds synergistically with the cell-surface protein CD36 to apoptotic cells through the bridging molecule thrombospondin, an extracellular matrix glycoprotein [50]. MFG-E8 binds to integrin α_v_ through its RGD domain. On the basis of amino acid sequence, PAT-2 is more closely related to the RGD-binding integrins than to the laminin-binding integrins. However, *C. elegans* does not appear to have an MFG-E8 homolog. A screen for RGD-containing molecules may be helpful in testing the involvement of RGD-containing molecules in the binding of PAT-2(ex)::mCherry to apoptotic cells.

Like PAT-2, CED-1 and INA-1 are expressed in muscle and hypodermal cells [40], [46]. It is intriguing that different receptors are preferentially used in different cell types when they are all present in these cells. This may be, in part, because some receptors preferentially bind to a subset, but not all, apoptotic cells. For example, PAT-2(ex)::mCherry binds to MSpppaaa, but not C1, C2 or C3, cell corpses (Figure S6A, S6C and S6D) and PAT-2 is required for the removal of MSpppaaa, but not C1, C2 or C3, cell corpses. In contrast, INA-1 and CED-1 receptors recognize apoptotic C1, C2 and C3 cells and mediate the engulfment of these cells [42], [46]. However, some cell corpses can be recognized by multiple receptors. For example, PAT-2(ex)::mCherry and INA-1(N)::GFP are co-localized on some apoptotic cells (Figure S6E). Therefore, additional factor(s) other than the receptor-cell corpse interaction determines the cell-type specificity of engulfment receptors. The observation that CDC-42 preferentially functions in muscle cells but not hypodermal cells for engulfment suggests that the downstream molecule(s) are important for the cell-type specificity of engulfment receptors. CED-10 and CDC-42 are important for the actin-based cytoskeleton rearrangement [72], which occurs during engulfment of apoptotic cells ([55], Figure 2C). Hypodermal cells and muscle cells appear to have different requirement for CED-10 and CDC-42 in cell corpse removal, although the two GTP ases are expressed in both cell types [76]. Hypodermal cells and muscle cells may utilize different actin-based phagocytosis mechanisms or employ different ways to activate the phagocytosis machinery for cell corpse removal.

We showed that the muscle-mediated internalization of cell corpses took apporximately 6 mintues (Figure 3A). Similarly, the internalization of the C3 cell corpse by the hypodermal cell ABplaapppp took about 6 minutes [42], [46]. However, previous studies by Wang *et al.* and Zou *et al.* [43], [53] showed that the time for the CED-1::GFP-mediated internalization of apoptotic cells may take about 18 or 25 minutes, respectively, although the identities of the engulfing cells are unclear. Therefore, the time necessary for the entire internalization process to occur from initiation to completion appears different for different engulfing cells. Nonetheless, at least some engulfment processes mediated by the muscle cell and hypodermal cell proceeds with similar kinetics.

Recently, conditional deletion of integrin α_v_ in the mouse immune system revealed that this protein is essential for the engulfment of apoptotic cells by gut-associated macrophages and dendritic cells [4]. In addition, mice lacking MFG-E8, which mediates apoptotic cell clearance through integrin α_v_, are defective in the removal of apoptotic B cells by tingible body macrophages in the spleen germinal centers [77]. However, little was previously known about whether integrin α or other engulfment receptors were involved in apoptotic cell removal mediated by amateur phagocytes, such as muscle cells. *C. elegans* provides a good system for studying the amateur phagocyte-mediated engulfment of apoptotic cells, as it does not have professional phagocytes. Our data showed that PAT-2 acts in muscle cells and transduces the engulfment signal through a novel signaling pathway for apoptotic cell removal. Recently, a mouse lacking ELMO1 showed a defect in Sertoli cell-mediated engulfment of apoptotic germ cells, but no engulfment defect was detected in macrophages or fibroblasts [31]. This result, together with our data, suggest that, like professional phagocytes [78], amateur phagocytes in different tissues utilize different sets of engulfment receptors and signaling molecules for apoptotic cell engulfment at the whole organism level.

## Materials and Methods

### Strains and alleles

The N2 Bristol strain was used as the reference wild-type strain. All strains were maintained on nematode growth medium (NGM) plates with *Escherichia coli* OP50 as the food source at 20°C unless otherwise noted [79]. The following mutations were used: linkage group I (LGI), *ced-1(e1735)*, *ced-12 (n3261, tp2)*; LGII, *cdc-42(gk388)*, *mIn1[mIs14 dpy-10(e128)]*; LGIII, *abi-1(ok640)*, *ced-4(n1162)*, *ced-6(n1813)*, *ced-7(n1892, n1996)*, *unc-79(e1068), pat-2(st567)*, *dpy-17(e164)*, *pat-3(st564)*, *qC1 dpy-19(e1259) glp-1(q339)*; LGIV, *ced-2(n1994)*, *ced-3(n717)*, *ced-5(n1812), ced-10(n1993, n3246, tm597)*; LGIV, *uig-1(ok884)*. *dpy-17(e164)* was used to balance *unc-79(e1068) pat-2(st567)*. The homozygous *unc-79(e1068) pat-2(st567)* mutants were also maintained using the extrachromosomal *P_pat-2_pat-2::gfp* or *P_pat-2_pat-2::mcherry* transgene. In either of the transgenic lines, the *unc-79(e1068) pat-2(st567)* mutant embryos that lost the transgene were both Pat and Ced. The numbers of cell corpses in the *unc-79(e1068)* mutant at the comma and 1.5-fold stages were similar to those of the wild-type at the same stage, indicating that *unc-79(e1068)* does not affect apoptosis. *qC1 dpy-19(e1259) glp-1(q339)* was used to balance *pat-3(st564)*. *mIn1* was used to balance *cdc-42(gk388)*. The *pat-2(st567)* allele has a G441D alternation in the extracellular domain. The information of the transgenic strains used in this work is listed in Table S4.

### Plasmid construction

Three vectors were used to generate constructs expressing fluorescent proteins, the *gfp* vector pPD95.75, the *mcherry* vector pYW806, and the *mrfp* vector pYW897. pYW806 and pYW897 were respectively generated by replacing *gfp* in pPD95.75 with *mcherry* or *mrfp* via the *Kpn*I/*Eco*RI sites. To generate *P_pat-2_pat-2::gfp* (pYW903) or *P_pat-2_pat-2::mCherry* (pYW950), the 10 kb fragment containing the 4 kb upstream sequence and the full-length *pat-2* coding region without the stop codon was PCR-amplified from the cosmid F54F2 (Sanger Institute, Cambridge, United Kingdom) using the primers TCCCCCCGGGTTTATGACTCACAGAC and GGGTACCGATGCATTTGTC CGTGACGT, and cloned into pPD95.75 or pYW806, respectively, via the *Kpn*I site. The full-length *pat-2* cDNA construct (pYW901) was generated by inserting into yk616b4 (Dr. Yuji Kohara) via the *Pst*I site a 0.6 kb *pat-2* cDNA which was amplified by RT-PCR using the primers AACTGCAGATGCGAGAGGGTAGTTTTCC and GATTCTTCTTTCCTGGAACTGCAGC. To generate *P_pat-2_nls::gfp* (pYW949) or *P_pat-2_gfp* (pYW903), the 4 kb upstream sequence of *pat-2* was first amplified by PCR from the cosmid F54F2 using the primers TCCCCCCGGGTTTATGACTCACAGAC and TCCCCCCGGGATCTACTGG AAATTTG and inserted into pPD95.67 or pPD95.75, respectively. The *P_unc-54_gfp* (pYW899) or *P_unc-54_mcherry* (pYW900) construct was generated by inserting the 1 kb *Hin*dIII/*Kpn*I fragment of pPD30.38 containing *P_unc-54_* into pPD95.75 or pYW806, respectively. The *P_ajm-1_gfp* (pYW902) was generated by inserting into pPD95.75 via the *Sal*I/*Bam*HI sites a 5.5 kb *P_ajm-1_* fragment which was amplified by PCR from pBR980 [69] using the primers CGTCGACCGATTTGACCGTTCGATAAG and CGGATCCTCGTCGGTA GTACTCGTCC. *P_ced-1_gfp* (pYW898) was generated by inserting into pPD95.75 via the *Sph*I site a 5 kb *P_ced-1_* fragment which was PCR-amplified from genomic DNA using the primers GGCATGCATACCTCCTGATATG GGGTGA and GCATGCTTGC GGCTGCAAAAAAACAGGG. *P_ced-1_ced-1::gfp* (pYW904) was generated by three-piece ligation. The 6 kb PCR-amplified fragment from *P_ced-1_ced-1::gfp* [40] using the primers AGGTACCATGCGTCTCATTCTCCTTGTGC and GGTCGA CGTGATTGTTCAGATGA and the 2.4 kb fragment PCR-amplified from genomic DNA using the primers CGTCGACCTCTATTAGAAGAGCATGACG and TGGTACCGAGGTGTACAAATTGTCCTGAGC were inserted into pYW898 via the *Sal*I and *Kpn*I sites. *P_ced-1_ced-1::gfp* fully rescued the engulfment defect of the *ced-1(e1735)* mutant (data not shown). To generate pYW901 containing *pat-2* cDNA without the stop codon, *pat-2* cDNA was PCR-amplified from the full-length *pat-2* cDNA clone using the primers CGGTACCATGCGAGAGGGTAGTTTTCC and GGGTACCGATAGCATTTGTC CGTGACGT and inserted into the pGEM-T Easy vector (Promega) via the *Kpn*I site. To generate *P_unc-54_pat-2::gfp* (pYW913) or *P_unc-54_pat-2::mCherry* (pYW916), the 3.7 kb *Kpn*I *pat-2* fragment from pYW901 was inserted into pYW899 or pYW900, respectively, via the *Kpn*I site. To generate *P_unc-54_ced-1::gfp* (pYW905), the 8.5 kb *Kpn*I *ced-1* fragment from pYW904 was inserted into pYW899 via the *Kpn*I site. To generate *P_unc-54_ ced-1::mrfp* (pYW941), a 8.6 kb *Spe*I/*Bam*HI fragment, containing *P_unc-54_* and the first 4.9 Kb of *ced-1*, and a 3.6 kb *Bam*HI/*Kpn*I fragment, corresponding to the rest *ced-1* sequence, of pYW905 were fused to a 1.5 kb *Kpn*I/*Spe*I fragment, containing the *mrfp* sequence, of pYW897 by three-piece-ligation. To generate *P_unc-54_moesin::gfp* (pYW940), the 0.6 kb *Sma*I/*Nco*I fragment containing the *moesin* actin-binding sequence was inserted into pYW899 via the *Nhe*I/*Nco*I sites. The *moesin* plasmid was a gift from Dr. Fabio Piano [75]. To generate *P_unc-54_ gfp::cdc-42* (pYW906), two plasmids gfp_Ntag_TA and cdc-42_Ntag_TA were generated first. To generate the gfp_Ntag_TA plasmid, PCR-amplified *gfp*, using oligonucleotides CGGTACCATGAGTAAAGGAG AAGAACT and GTCTAGATTTGTATAGTTCATCCATGCC as primers and pPD95.75 as template was inserted to the vector pGEM T-Easy. To generate the cdc-42_Ntag_TA plasmid, PCR-amplified *cdc-42* from k1101h01(Y. Kohara) using primers GTCTAGAATGCAGACGATCAAGTGCG and CGTTAACCTAGAGAATATTGCACTTCTTC was inserted into the pGEM T-Easy vector. The 1 kb *Hin*dIII/*Kpn*I fragment of pPD30.38 containing *P_unc-54_*, the *Kpn*I /*Xba*I gfp fragment from the gfp_Ntag_TA plasmid, and the *Xba*I/*Eco*R1 *cdc-42* fragment from the cdc-42_Ntag_TA plasmid were inserted to the pPD95.75 vector previously digested with *Hind*III and *Eco*RI in a four-piece-ligation reaction to generate pYW906. To generate *P_ajm-1_pat-2::gfp* (pYW948) or *P_ajm-1_ced-1::gfp* (pYW914), the 3.7 kb *Kpn*I *pat-2* sequence from pYW901 or the 8.5 kb *Kpn*I *ced-1* sequence from pYW904 was inserted, respectively, into pYW902. To generate *P_ced-1_pat-2::gfp* (pYW942), the 3.7 kb *Kpn*I *pat-2* sequence from pYW901 was inserted into pYW898 via the *Kpn*I site. To generate *P_pat-2_pat-2Δcyto::gfp* (pYW964), *pat-2Δcyto* was PCR-amplified from pYW901 using the primers CGGTACCATGCGA GAGGGTAGTTTTCC and TTGGT ACCGTC CTATAGAATAATGCAA and cloned into pYW903 via the *Kpn*I site. To generate *Pemphtype*pat-2(ex)::*mcherry* (pYW917), the DNA fragment corresponding to *pat-2(ex)* was PCR-amplified from pYW901 using the primers CGGTACCATGCGAGAGG GTAGTTTTCC and CGGTACCAGATCTCTTCC TTCTTCAGA and cloned into pYW900 via the *Kpn*I site. To generate *P_hsp_pat-2(ex)::mcherry* (pYW966), the 4.2 kb *Nhe*I/*Pvu*I fragment of the *P_unc-54_ pat-2(ex)::mCherry* plasmid containing *pat-2(ex)::mCherry* was inserted into pPD49.78 via the *Nhe*I/*Pvu*I sites. To generate *P_hsp_gfp::cdc-42* (pYW959), the 1.5 kb *Kpn*I/*Hpa*I fragment of pYW906 corresponding to *gfp::cdc-42* was inserted into pPD49.78 and pPD49.83 (different tissue specificity) via the *Kpn*I/*Eco*RV sites. To generate the *P_unc-54_ina-1::gfp* plasmid, *P_unc-54_* was PCR-amplified using pYW913 as template and oligonucleotides GCATCCGCCAAGCTTGTCTTCTTC and GGATCCGGTACCGT CGACGCTAC as primers and used to replace the *P_ced-1_* region of *P_ced-1_ina-1::gfp* via the *Sph*I/*BamH*I sites. To generate the *P_pat-3_pat-3::gfp* (pYW1091) plasmid, the 10.2 kb *pat-3* genomic DNA was amplified by PCR and subsequently cloned into the pPD95.75 vector via the *Xma*I/*Kpn*I sites. To generate the *P_unc-54_myri::mrfp* (pYW1092) construct, the *myri::mrfp* cDNA of *P_unc-86_myri::gfp* (from C. Bargmann) was inserted into the *P_unc-54_mrfp* vector via the *Kpn*I site. To generate the *P_ajm-1_gfp::cdc-42* (pYW1093) plasmid, the gfp region of the pPD95.75 plasmid was replaced by *gfp::cdc-42* from the *P_hsp_gfp::cdc-42* construct via the *Kpn*I site, and the resulting construct was then digested with *Bam*HI and *Sal*I and subsequently inserted with the *Bam*HI and *Sal*I fragment containing the P*_ajm-1_* promoter from pYW902.

### RNAi experiments

To generate the *pat-2* RNAi clone, the 1.3 kb *Pst*I/*Hind*III fragment of *pat-2* cDNA was inserted into the pPD129.36 vector. The *pat-3* RNAi plasmid was obtained from the J. Ahringer RNAi library. All RNAi experiments were carried out using a bacterial feeding protocol [80]. In brief, L4 larvae were laid out on control plates (pPD129.36) or the indicated RNAi plates and cultured at 20°C for 48 h, then the F1 embryos were picked for phenotypic analysis.

### Transgenic animals

Transgenic animals were generated by microinjection of the indicated plasmid(s) (3–50 ng/µL), using the pRF4[*rol-6 (su1006)*], pTG96[*sur-5::gfp*] or *P_sur-5_rfp* plasmids (50 ng/µL) as coinjection markers [81], [82]. The injection procedure was performed as described previously [8]. The resulting transgenes and genetic backgrounds of the strains were listed in Table S4. *P_hsp_ced-10V12* was injected with the coinjection marker pTG96[*sur-5::gfp*] to *unc-79(e1068) pat-2(st567)/dpy-17(e164)*, and no rescue of the Pat phenotype was observed. To score cell corpses, transgenic embryos carrying the transgene *P_hsp_ced-10V12* and pTG96[*sur-5::gfp*] were scored and only the data for those which later exhibited the Pat phenotype were used.

### Microscopy

Embryos were mounted on a 4% agar pad in M9 buffer in the presence (four-fold stage embryos) or absence (comma and 1.5-fold stage embryos) of 30 mM sodium azide at 20°C. Cell corpses were analyzed using DIC microscopy, as previously described [46]. A Zeiss Axioplan 2 microscope equipped with a digital camera (AxioCam; Carl Zeiss, Inc.) and 4.7 AxioVision imaging software was used. To obtain the cell corpse data of homozygous *pat-2(st567)* embryos derived from the heterozygous *unc-79(e1068) pat-2(st567)/dpy-17(e164)* mothers, embryos at the indicated developmental stages were analyzed and only the data for those which later exhibited the Pat phenotype were used. To obtain the cell corpse data of homozygous *pat-2(st567)* embryos derived from *unc-79(e1068) pat-2(st567)* mothers carrying either *P_pat-2_pat-2::gfp* or *P_pat-2_pat-2::mcherry* transgene, non-fluorescent embryos at the indicated developmental stages were analyzed. To obtain the cell corpse data of homozygous *cdc-42(gk388)* embryos derived from the heterozygous *cdc-42(gk388)/mIn1[mIs14 dpy-10(e128)]* mothers, GFP-negative embryos at the indicated developmental stages were analyzed. To analyze cell death events during embryogenesis, wild-type or mutant embryos were filmed during the period 200–460 min after the first cleavage and the time point at which each cell corpse appeared was noted and was reported relative to the first cell death for comparison between the wild-type and mutants. About 40–50 serial Z-sections were recorded at 0.4 µm intervals every 1 min. To measure the duration of cell corpses in the wild-type and mutants, cell corpses appearing between 360 to 410 min after the first cleavage during embryogenesis were followed, and about 50–60 serial Z-sections were recorded at 0.3 or 0.4 µm intervals every 1 min. To monitor the muscle-mediated internalization of apoptotic cells, the fluorescence images of wild-type or mutant embryos carrying the *P_unc-54_myri::mrfp* transgene were recorded using the DeltaVision microscope (GE Healthcare company) equipped with a digital camera (Photometrics Cascade II 512 EMCCD) at 1 (for wild-type) or 3 (for mutant)-minute intervals for about 120 minutes.

### Heat shock experiments

For the heat shock rescue experiments, transgenic embryos were subjected to heat shock at 33°C for 30 min and transferred to 20°C to recover for 2 hours, then cell corpses in embryos at the indicated stages were counted. To test the binding of PAT-2(ex)::mCherry to apoptotic cells and its effect on cell-corpse engulfment when overexpressed, embryos carrying the transgene *P_hsp_pat-2(ex)*::*mcherry* or embryos carrying the transgenes *P_hsp_pat-2(ex)*::*mcherry* and *P_hsp_ina-1(N)*::*gfp* were subjected to heat shock at 33°C for 60 min and transferred to 20°C to recover for 4–5 hours, then embryos were examined using fluorescence microscopy (for mCherry and/or INA-1(N)::GFP signal) or DIC microscopy (for cell corpses). To overexpress GFP::CDC-42, CED-10V12 or Annexin V::mRFP by heat-shock, the embryos carrying the respective transgene were subjected to heat shock at 33°C for 30 min and transferred to 20°C to recover for 2 hours, then embryos were examined using fluorescence microscopy (mCherry signal) or DIC microscopy (cell corpses).

## Supporting Information

Figure S1 *pat-2* expression is not detectable in embryonic apoptotic cells. (A) GFP, Annexin V::mRFP, merged GFP and mRFP, and DIC images of a wild-type early embryo co-expressing *P_pat-2_nls::gfp* and *P_hsp_annexin V::mrfp* transgenes after heat shock treatment. Annexin V::mRFP, which is secreted and clusters around the surface of apoptotic cells, was used to label apoptotic cells. Apoptotic cells are indicated by arrows. The scale bar represents 5 µm. (B) GFP, Annexin V::mRFP, merged GFP and mRFP, and DIC images of a wild-type late embryo co-expressing *P_pat-2_nls::gfp* and *P_hsp_annexin V::mrfp* transgenes after heat shock treatment. Apoptotic MSpppaaa cells are indicated by arrows. The scale bar represents 5 µm.
(TIF)

Figure S2 PAT-2 and PAT-3 are co-localized in muscle cells and apoptotic cells. A wild-type larva (A) and an embryo (B) co-expressing the transgenes *P_pat-2_pat-2::mcherry* and *P_pat-3_pat-3::gfp*. (A) PAT-2::mCherry and PAT-3::GFP were co-localized to the dense bodies (arrows) and M-lines (arrowheads) in muscle cells. The scale bar represents 10 µm. (B) PAT-2::mCherry and PAT-3::GFP were co-localized around an apoptotic cell (arrows). The scale bar represents 5 µm.
(TIF)

Figure S3 PAT-2::GFP localization around cell corpses is not disrupted by either *ced-1* or *ced-5* mutation. PAT-2::GFP (left) and DIC (right) images of a *ced-1(e1735); ced-5(n1812)* embryo carrying the transgene *P_pat-2_pat-2::gfp*. Apoptotic cells are indicated by arrows and shown enlarged in insets. The scale bar represents 5 µm.
(PDF)

Figure S4 Expression of *P_unc-54_nls::gfp* or *P_unc-54_pat-2(ex)::mcherry* transgene in embryos. (A) DIC and GFP images of a *ced-1(e1735)* mutant embryo expressing *P_unc-54_nls::gfp*. Arrows indicate apoptotic cells. (B) DIC and PAT-2(ex)::mCherry images of a wild-type embryo expressing *P_unc-54_pat-2(ex)::mcherry*. Arrows indicate MSpppaaa cells. Both scale bars represent 5 µm.
(TIF)

Figure S5 The MSpppaaa cell corpse is engulfed by a muscle cell. PAT-2::GFP, PAT-2::mCherry, and DIC images of an embryo co-expressing *P_ajm-1_pat-2::gfp* and *P_unc-54_pat-2::mcherry*. MSpppaaa cell corpses are indicated by arrows and shown enlarged in insets. The scale bar represents 5 µm.
(TIF)

Figure S6 Localization of PAT-2 (ex)::mCherry, CED-1::GFP and INA-1(N)::GFP around apoptotic cells. (A) Localization of PAT-2 (ex)::mCherry around the apoptotic MSpppaaa cell. DIC and PAT-2 (ex)::mCherry images of a wild-type embryo expressing *P_hsp_pat-2(ex)::mcherry* at the time of pharyngeal pumping. Apoptotic MSpppaaa cells are indicated by arrows. (B) Localization of CED-1::GFP around the apoptotic MSpppaaa cell. DIC and CED-1::GFP images of a wild-type embryo expressing *P_ced-1_ced-1::gfp*. (C, D) The PAT-2(ex)::mCherry signal is not observed around C1, C2 and C3 cell corpses. DIC and PAT-2 (ex)::mCherry images of *ced-1(e1735); ced-5(n1812)* double mutant embryos expressing the transgene *P_hsp_pat-2(ex)::mcherry*. C1, C2 and C3 cell corpses are indicated by arrows. (E) Co-localization of PAT-2 (ex)::mCherry and INA-1(N)::GFP on some apoptotic cells. DIC, PAT-2 (ex)::mCherry, INA-1(N)::GFP and merged images of a *ced-1(e1735); ced-5(n1812)* double mutant embryo co-expressing the transgenes *P_hsp_pat-2(ex)::mcherry* and *P_hsp_ina-1(N)::gfp*. Apoptotic cells are indicated by arrows. All scale bars represent 5 µm.
(TIF)

Figure S7 Deletion of the cytoplasmic domain of PAT-2 does not affect its localization to muscle cell surfaces, dense bodies and M-lines. (A) The DIC and PAT-2Δcyto::GFP images of wild-type and *pat-2(st567)* adult worms expressing the transgene *P_pat-2_pat-2Δcyto::gfp*. PAT-2Δcyto::GFP is localized to muscle cell boundaries (indicated by open arrowheads, outlined in the right panel), dense bodies (indicated by arrows) and M-lines (indicated by close arrowheads). The scale bar represents 10 µm. (B–C) PAT-2::GFP and PAT-2Δcyto::GFP are localized to the surface of muscle precursor cells. The DIC, PAT-2Δcyto::GFP (B) and PAT-2::GFP (C) images of wild-type and *pat-2(st567)* embryos carrying the *P_unc-54_pat-2Δcyto::gfp* transgene (B) or the wild-type carrying the *P_pat-2_pat-2::gfp* transgene (C). All scale bars represent 5 µm.
(TIF)

Figure S8 The internalization of MSpppaaa cell corpses is defective in the *pat-2(st567); Ex[P_pat-2_pat-2Δcyto::gfp]* embryos. The DIC and PAT-2Δcyto::GFP images of a wild-type and a *pat-2(st567)* embryos expressing *P_pat-2_pat-2Δcyto::gfp* at the stage when grinder formation had finished. A PAT-2Δcyto::GFP circle was detected around the MSpppaaa cell corpse (indicated by an arrow) in the wild-type (A) but not in the *pat-2(st567)* (B) embryo. The engulfing muscle cell for the MSpppaaa cell corpse is indicated by an arrowhead. The scale bar represents 5 µm.
(PDF)

Table S1 Localization of PAT-2 or PAT-2(ex) around apoptotic cells.
(DOC)

Table S2 The engulfment of C1, C2 and C3 cell corpses appears normal in *pat-2* and *cdc-42* mutant embryos.
(DOC)

Table S3 Effects of *pat-2Δcyto::gfp* on the Ced phenotype during mid and late embryogenesis.
(DOC)

Table S4 List of transgenic strains and transgenes used in this work.
(DOC)

Text S1 *pat-2* does not appear to be expressed in embryonic apoptotic cells.
(RTF)
