# Supplementary figures and images for: Integrin α PAT-2/CDC-42 Signaling Is Required for Muscle-Mediated Clearance of Apoptotic Cells in Caenorhabditis elegans
Source: PLoS Genet. 2012 May 17;8(5):e1002663. doi: 10.1371/journal.pgen.1002663 (PMC3355063; doi:10.1371/journal.pgen.1002663)

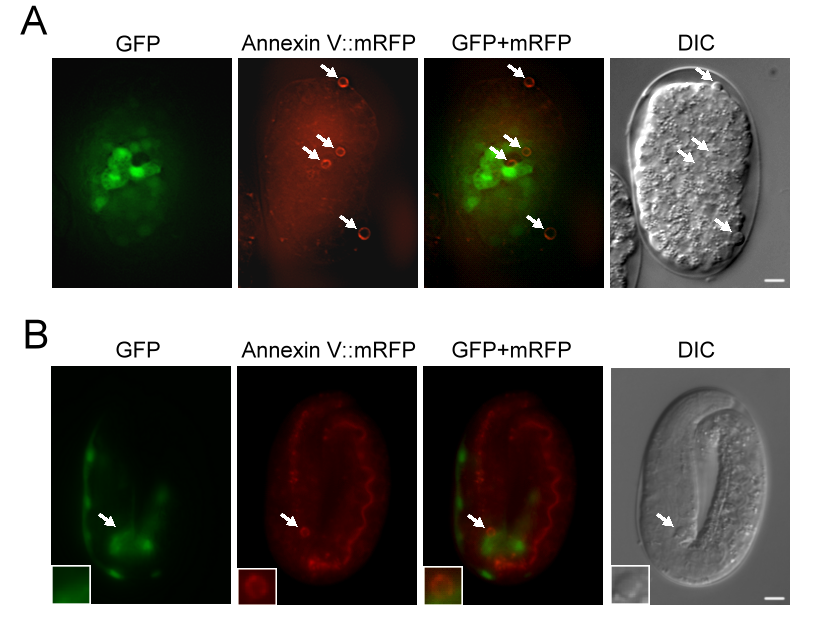

Supplement: Figure S1 — pat-2 expression is not detectable in embryonic apoptotic cells. (A) GFP, Annexin V::mRFP, merged GFP and mRFP, and DIC images of a wild-type early embryo co-expressing Ppat-2nls::gfp and Phspannexin V::mrfp transgenes after heat shock treatment. Annexin V::mRFP, which is secreted and clusters around the surface of apoptotic cells, was used to label apoptotic cells. Apoptotic cells are indicated by arrows. The scale bar represents 5 µm. (B) GFP, Annexin V::mRFP, merged GFP and mRFP, and DIC images of a wild-type late embryo co-expressing Ppat-2nls::gfp and Phspannexin V::mrfp transgenes after heat shock treatment. Apoptotic MSpppaaa cells are indicated by arrows. The scale bar represents 5 µm. (TIF) [file pgen.1002663.s001.tif]

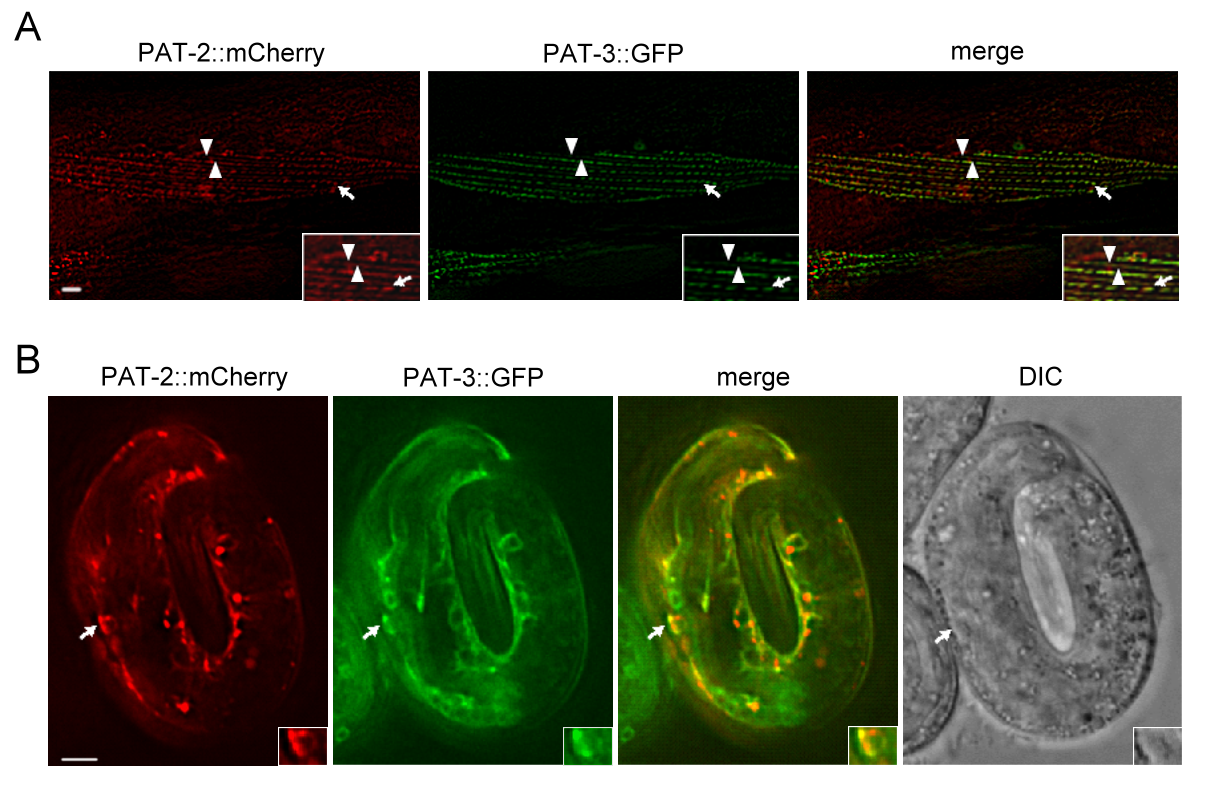

Supplement: Figure S2 — PAT-2 and PAT-3 are co-localized in muscle cells and apoptotic cells. A wild-type larva (A) and an embryo (B) co-expressing the transgenes Ppat-2pat-2::mcherry and Ppat-3pat-3::gfp. (A) PAT-2::mCherry and PAT-3::GFP were co-localized to the dense bodies (arrows) and M-lines (arrowheads) in muscle cells. The scale bar represents 10 µm. (B) PAT-2::mCherry and PAT-3::GFP were co-localized around an apoptotic cell (arrows). The scale bar represents 5 µm. (TIF) [file pgen.1002663.s002.tif]

Figure S3

PAT-2::GFP

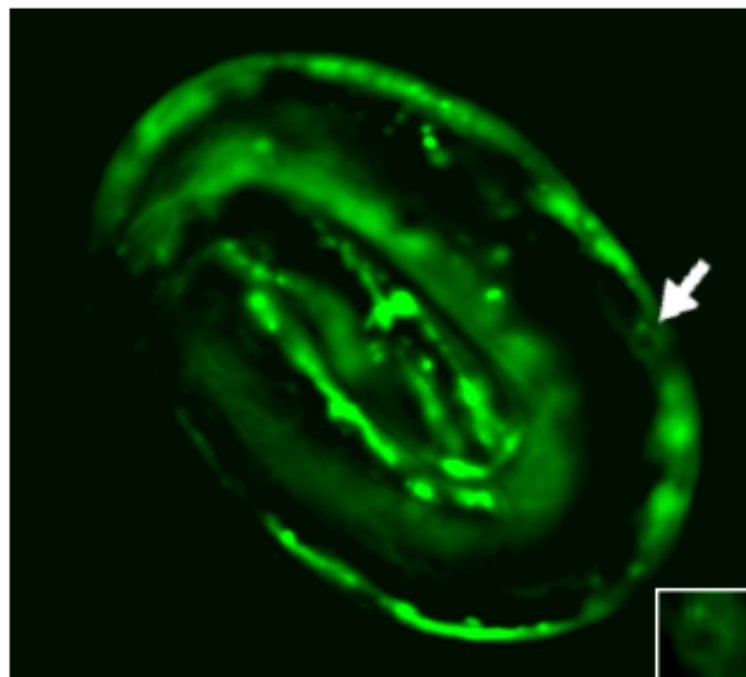

DIC

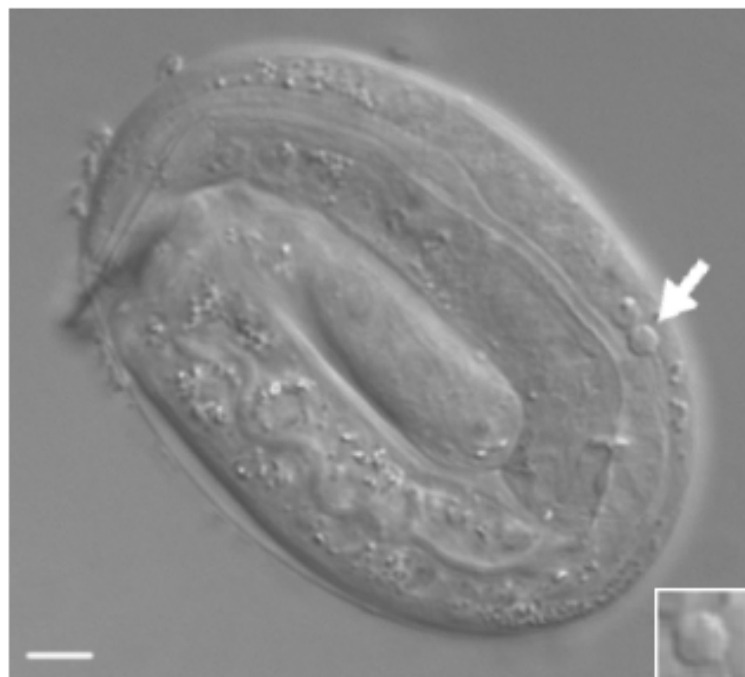

Supplement: Figure S3 — PAT-2::GFP localization around cell corpses is not disrupted by either ced-1 or ced-5 mutation. PAT-2::GFP (left) and DIC (right) images of a ced-1(e1735); ced-5(n1812) embryo carrying the transgene Ppat-2pat-2::gfp. Apoptotic cells are indicated by arrows and shown enlarged in insets. The scale bar represents 5 µm. (PDF) [file pgen.1002663.s003.pdf]

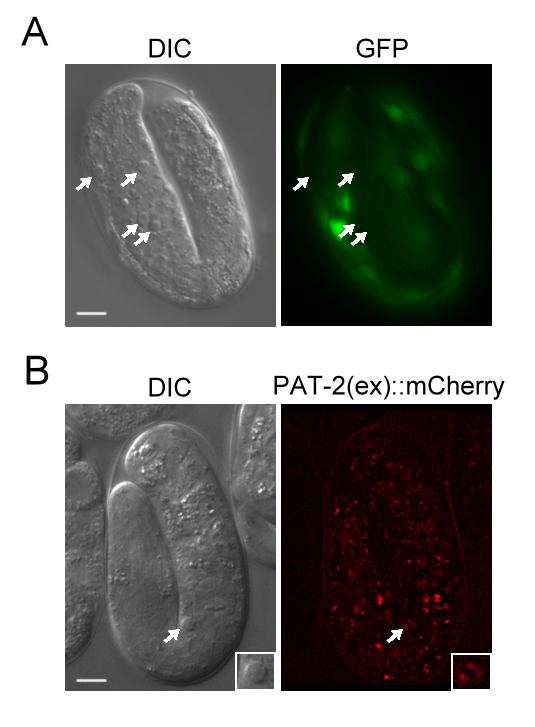

Supplement: Figure S4 — Expression of Punc-54nls::gfp or Punc-54pat-2(ex)::mcherry transgene in embryos. (A) DIC and GFP images of a ced-1(e1735) mutant embryo expressing Punc-54nls::gfp. Arrows indicate apoptotic cells. (B) DIC and PAT-2(ex)::mCherry images of a wild-type embryo expressing Punc-54pat-2(ex)::mcherry. Arrows indicate MSpppaaa cells. Both scale bars represent 5 µm. (TIF) [file pgen.1002663.s004.tif]

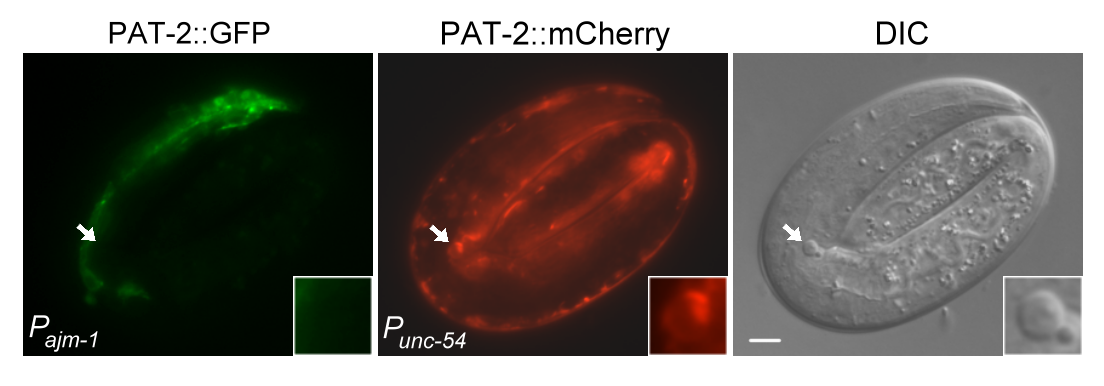

Supplement: Figure S5 — The MSpppaaa cell corpse is engulfed by a muscle cell. PAT-2::GFP, PAT-2::mCherry, and DIC images of an embryo co-expressing Pajm-1pat-2::gfp and Punc-54pat-2::mcherry. MSpppaaa cell corpses are indicated by arrows and shown enlarged in insets. The scale bar represents 5 µm. (TIF) [file pgen.1002663.s005.tif]

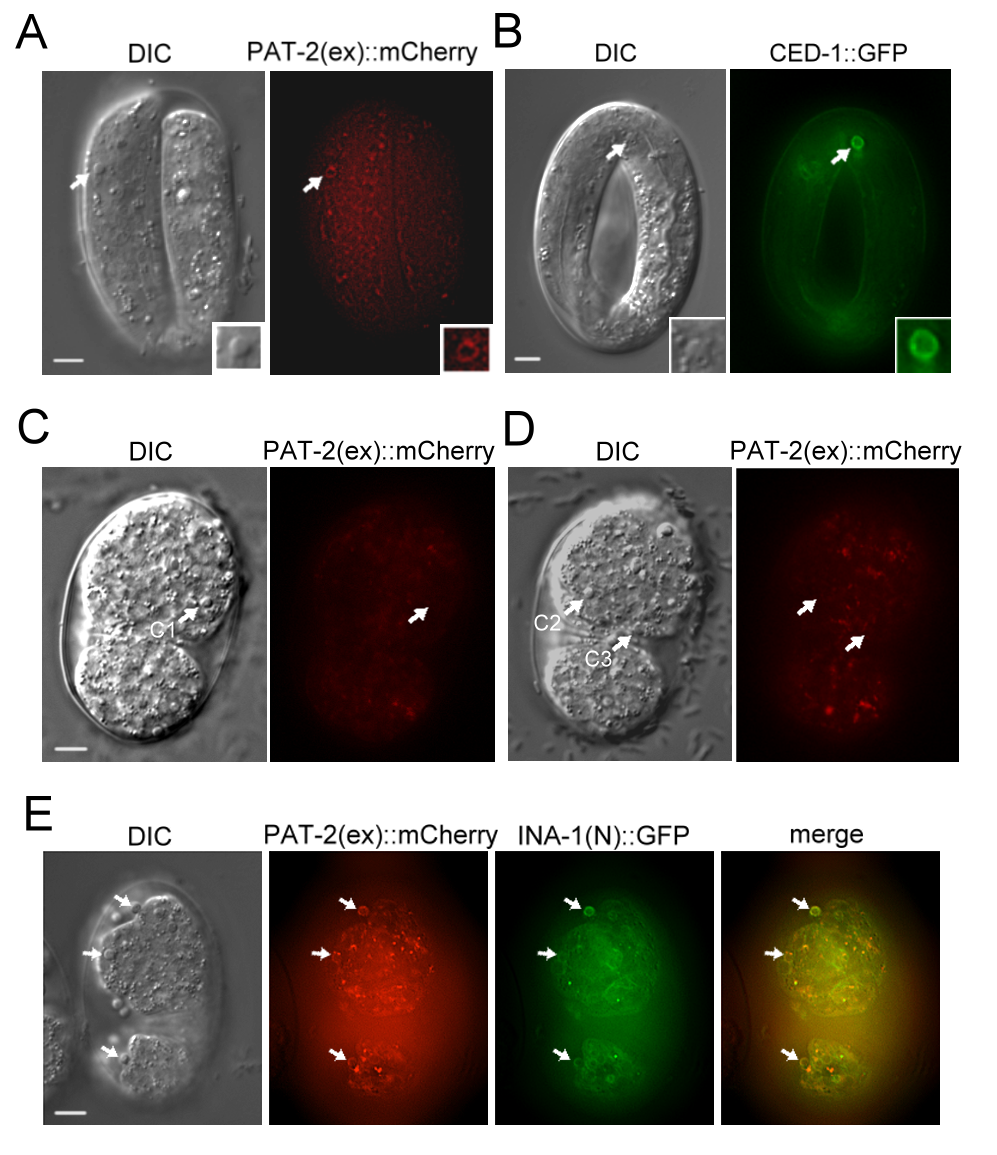

Supplement: Figure S6 — Localization of PAT-2 (ex)::mCherry, CED-1::GFP and INA-1(N)::GFP around apoptotic cells. (A) Localization of PAT-2 (ex)::mCherry around the apoptotic MSpppaaa cell. DIC and PAT-2 (ex)::mCherry images of a wild-type embryo expressing Phsppat-2(ex)::mcherry at the time of pharyngeal pumping. Apoptotic MSpppaaa cells are indicated by arrows. (B) Localization of CED-1::GFP around the apoptotic MSpppaaa cell. DIC and CED-1::GFP images of a wild-type embryo expressing Pced-1ced-1::gfp. (C, D) The PAT-2(ex)::mCherry signal is not observed around C1, C2 and C3 cell corpses. DIC and PAT-2 (ex)::mCherry images of ced-1(e1735); ced-5(n1812) double mutant embryos expressing the transgene Phsppat-2(ex)::mcherry. C1, C2 and C3 cell corpses are indicated by arrows. (E) Co-localization of PAT-2 (ex)::mCherry and INA-1(N)::GFP on some apoptotic cells. DIC, PAT-2 (ex)::mCherry, INA-1(N)::GFP and merged images of a ced-1(e1735); ced-5(n1812) double mutant embryo co-expressing the transgenes Phsppat-2(ex)::mcherry and Phspina-1(N)::gfp. Apoptotic cells are indicated by arrows. All scale bars represent 5 µm. (TIF) [file pgen.1002663.s006.tif]

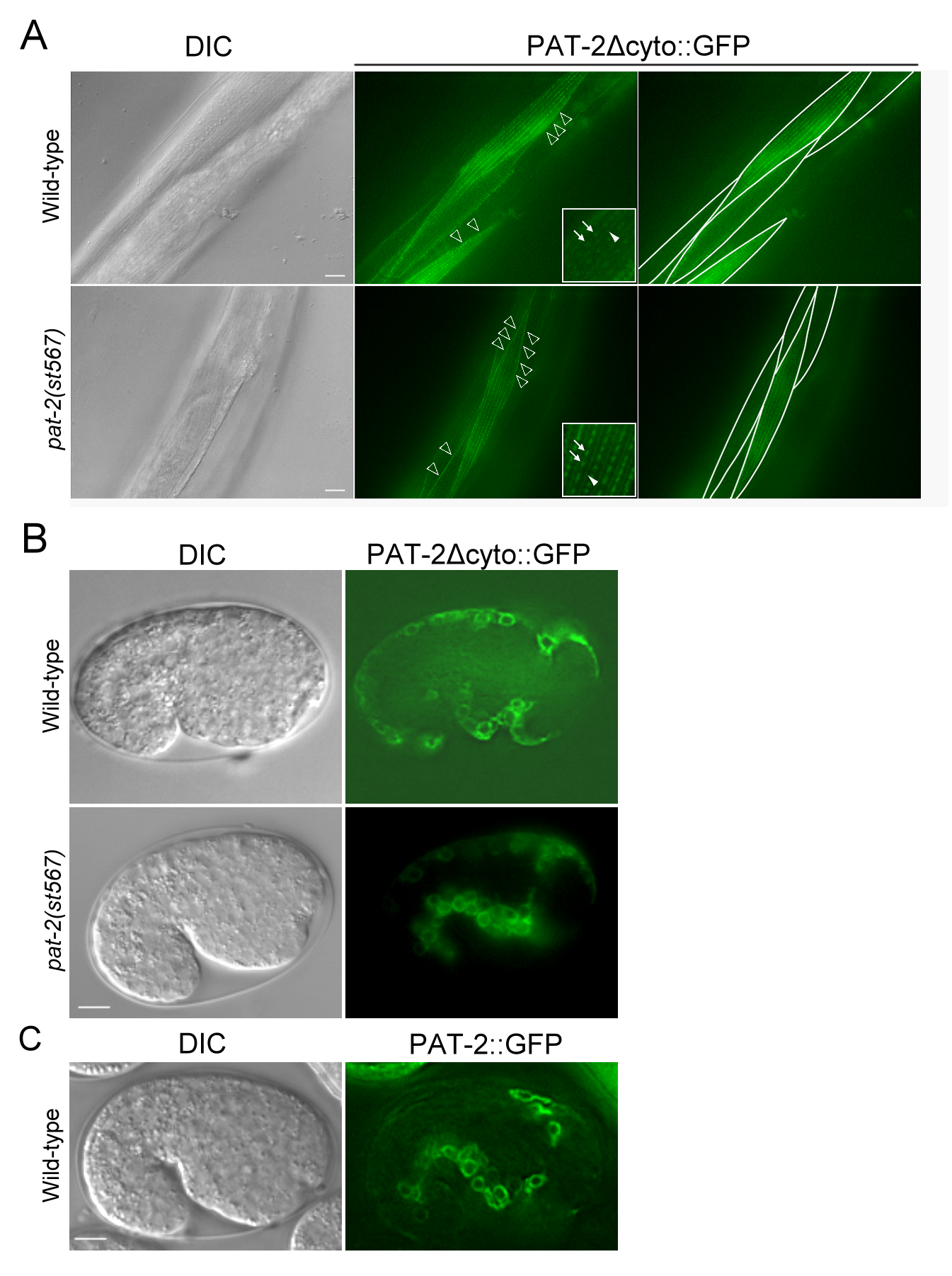

Supplement: Figure S7 — Deletion of the cytoplasmic domain of PAT-2 does not affect its localization to muscle cell surfaces, dense bodies and M-lines. (A) The DIC and PAT-2Δcyto::GFP images of wild-type and pat-2(st567) adult worms expressing the transgene Ppat-2pat-2Δcyto::gfp. PAT-2Δcyto::GFP is localized to muscle cell boundaries (indicated by open arrowheads, outlined in the right panel), dense bodies (indicated by arrows) and M-lines (indicated by close arrowheads). The scale bar represents 10 µm. (B–C) PAT-2::GFP and PAT-2Δcyto::GFP are localized to the surface of muscle precursor cells. The DIC, PAT-2Δcyto::GFP (B) and PAT-2::GFP (C) images of wild-type and pat-2(st567) embryos carrying the Punc-54pat-2Δcyto::gfp transgene (B) or the wild-type carrying the Ppat-2pat-2::gfp transgene (C). All scale bars represent 5 µm. (TIF) [file pgen.1002663.s007.tif]

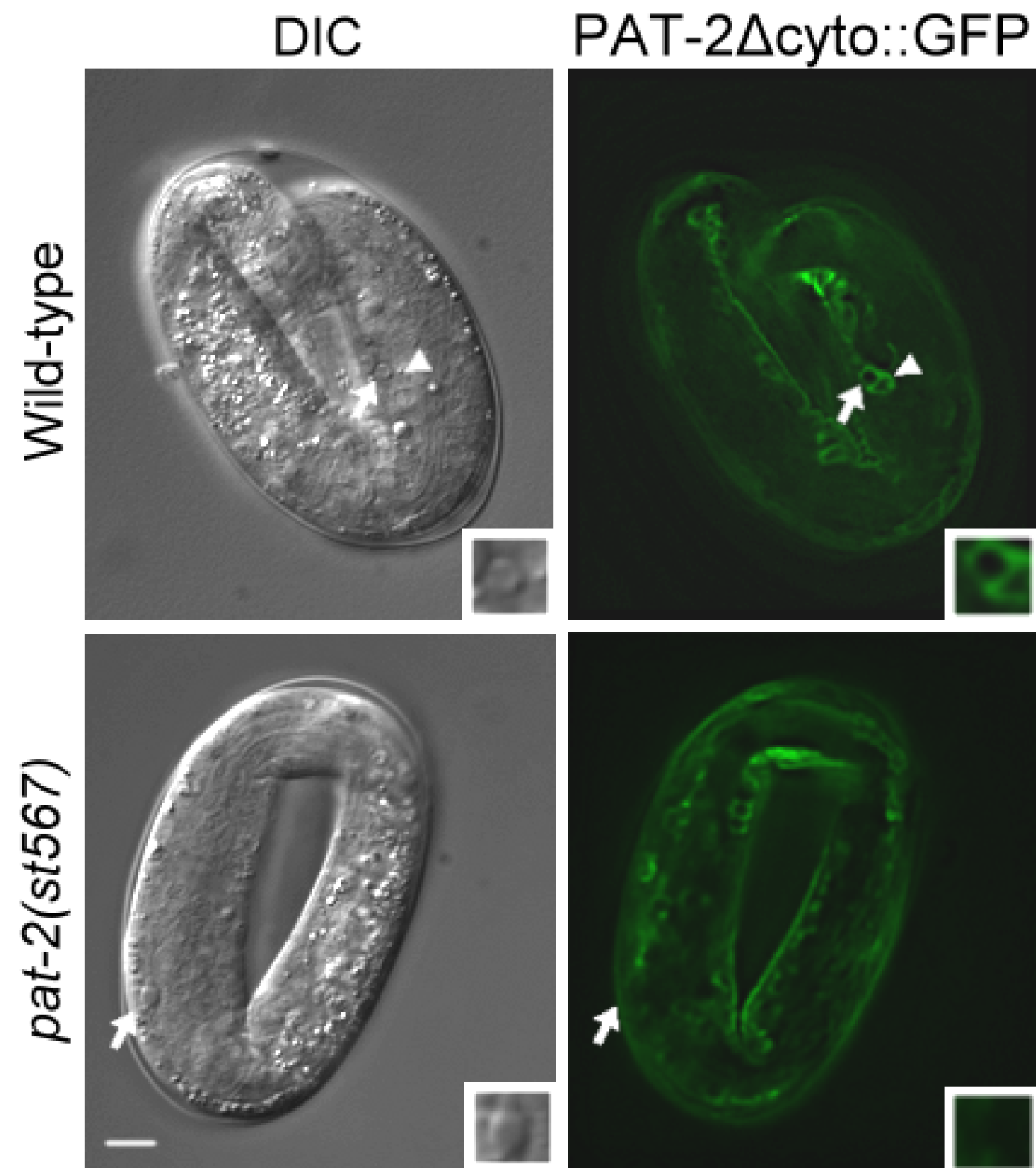

Supplement: Figure S8 — The internalization of MSpppaaa cell corpses is defective in the pat-2(st567); Ex[Ppat-2pat-2Δcyto::gfp] embryos. The DIC and PAT-2Δcyto::GFP images of a wild-type and a pat-2(st567) embryos expressing Ppat-2pat-2Δcyto::gfp at the stage when grinder formation had finished. A PAT-2Δcyto::GFP circle was detected around the MSpppaaa cell corpse (indicated by an arrow) in the wild-type (A) but not in the pat-2(st567) (B) embryo. The engulfing muscle cell for the MSpppaaa cell corpse is indicated by an arrowhead. The scale bar represents 5 µm. (PDF) [file pgen.1002663.s008.pdf]
