# Supplementary material for: Integrin α PAT-2/CDC-42 Signaling Is Required for Muscle-Mediated Clearance of Apoptotic Cells in Caenorhabditis elegans
Source: PLoS Genet. 2012 May 17;8(5):e1002663. doi: 10.1371/journal.pgen.1002663 (PMC3355063; doi:10.1371/journal.pgen.1002663)
Supplement: Table S1 — Localization of PAT-2 or PAT-2(ex) around apoptotic cells. (DOC) [file pgen.1002663.s009.doc]

**Supporting Tables**

| **Table S1. Localization of PAT-2 or PAT-2(ex) fusion proteins around apoptotic cells** | | | | |
| --- | --- | --- | --- | --- |
| Genotype | | Transgene | % corpses with mCherry circle*^a^* % corpses with GFP circle | |
| Corpses(1.5-fold) MSpppaaa C1,C2,C3 | at 1.5-fold stage |
| Wild-type*^b^* | *P_pat-2_pat-2::mcherry* and  *P_unc-54_pat-2::gfp* | | 22.7 ND ND | 18.1 |
| Wild-type  /heatshock | *P_hsp_pat-2(ex)::mcherry* | | 16.6 20.0 0.0 | - |
| *^a^*Percentage of indicated cell corpses (corpses in the 1.5-fold embryos, MSpppaaa, or C1, C2 and C3) labeled with the mCherry circle. *^b^*Wild-type embryos co-expressing *P_pat-2_pat-2::mcherry* and *P_unc-54_pat-2::gfp* transgenes. N.D: not determined. -: not applicable. The transgenic worms were generated as described in [Materials and Methods](http://www.plosgenetics.org/static/guidelines.action#materials_methods%23materials_methods). | | | | |
