# Supplementary material for: Integrin α PAT-2/CDC-42 Signaling Is Required for Muscle-Mediated Clearance of Apoptotic Cells in Caenorhabditis elegans
Source: PLoS Genet. 2012 May 17;8(5):e1002663. doi: 10.1371/journal.pgen.1002663 (PMC3355063; doi:10.1371/journal.pgen.1002663)
Supplement: Table S2 — The engulfment of C1, C2 and C3 cell corpses appears normal in pat-2 and cdc-42 mutant embryos. (DOC) [file pgen.1002663.s010.doc]

| **Table S2. The engulfment of C1, C2 and C3 cell corpses appears normal in *pat-2* and *cdc-42* mutant embryos.** | | | |
| --- | --- | --- | --- |
| Genotype | Duration time of cell corpses (min) *^a^* | | |
|  | C1 | C2 | C3 |
| Wild-type | 27.0 ± 17.4 (14-64) | 25.1 ± 6.9 (20-36) | 21.3 ± 7.4 (16-36) |
| *unc-79(e1068)* | 22.5 ± 6.8 (16-32) | 24.0 ± 6.5 (16-32) | 24.5 ± 4.7 (18-28) |
| *unc-79(e1068) pat-2(st567)^b^* | 30.3 ± 5.0 (25-35) | 39.7 ± 17.6 (23-58) | 36.7 ± 10.3 (28-48) |
| *cdc-42(gk388)^c^* | 26.0 ± 10.6 (14-34) | 28.0 ± 14.5 (12-46) | 30.0 ± 10.6 (22-42) |
| *^a^*The duration time of C1, C2 and C3 cell corpses was measured by minutes using time-lapse DIC microscopy. Data are presented as mean ± standard deviation, with range in parentheses. *^b^*Non-transgenic progeny of *unc-79(e1068) pat-2(st567); Ex[P_pat-2_pat-2::gfp]. ^c^*Homozygous progeny of *cdc-42(gk388)/mIn1[mIs14 dpy-10(e128)].* Statistical analysis was performed by the unpaired t test. There is no significant difference between wild-type and *cdc-42(gk388)* or *unc-79(e1068)* and *unc-79(e1068) pat-2(st567)*. The number of embryos scored: wild-type, 9; *unc-79(e1068)*, 4; *unc-79(e1068) pat-2(st567)*, 3; *cdc-42(gk388)*, 4. | | | |
