# Supplementary material for: Integrin α PAT-2/CDC-42 Signaling Is Required for Muscle-Mediated Clearance of Apoptotic Cells in Caenorhabditis elegans
Source: PLoS Genet. 2012 May 17;8(5):e1002663. doi: 10.1371/journal.pgen.1002663 (PMC3355063; doi:10.1371/journal.pgen.1002663)
Supplement: Table S3 — Effects of pat-2Δcyto::gfp on the Ced phenotype during mid and late embryogenesis. (DOC) [file pgen.1002663.s011.doc]

| **Table S3. Effects of** *pat-2Δcyto::gfp* **on the Ced phenotype during mid and late embryogenesis** | | | |
| --- | --- | --- | --- |
| Genotype | Transgene | No. of cell corpses ^a^ | |
| 2-fold | 3-fold 4-fold |
| Wild-type | *-* | 8.3 ± 0.7 | 2.5 ± 0.4 0.3 ± 0.5 |
| Wild-type | *P_pat-2_pat-2Δcyto::gfp* | 11.0 ± 0.7^**^ | 4.4 ± 0.8^**^ 2.3 ± 0.4^**^ |
| *pat-2(st567)* | *P_pat-2_pat-2Δcyto::gfp* | 12.5 ± 0.5^**^ | 4.8 ± 0.7^**^ 3.1 ± 0.5^**^ |
| *ced-1(e1735)* | *P_pat-2_ced-1::gfp* | 18.0 ± 4.0^**^ | 5.8 ± 2.1^**^ 2.3 ± 0.7^**^ |
| *^a^*Percentage of embryos with the MSpppaaa cell corpse at the indicated developmental stage. The transgenic worms were generated as described in [Materials and Methods](http://www.plosgenetics.org/static/guidelines.action#materials_methods%23materials_methods). Strains carrying the transgene were compared to the wild-type without the transgene (** *p* <0.001). The data are presented as the mean ± standard deviation (SD), n>10. | | | |
