# Supplementary material for: Integrin α PAT-2/CDC-42 Signaling Is Required for Muscle-Mediated Clearance of Apoptotic Cells in Caenorhabditis elegans
Source: PLoS Genet. 2012 May 17;8(5):e1002663. doi: 10.1371/journal.pgen.1002663 (PMC3355063; doi:10.1371/journal.pgen.1002663)
Supplement: Table S4 — List of transgenic strains and transgenes used in this work. (DOC) [file pgen.1002663.s012.doc]

| **Table S4. List of transgenic strains and transgenes used in this work** | | |
| --- | --- | --- |
| Strain | Transgenes | Genetic background |
| YW666 | tpEx288[*P_pat-2_pat-2::gfp;* rol-6*(*su1006*)]* | *unc-79(e1068) pat-2(st567)* |
| YW745 | *tpEx335[P_pat-2_pat-2::mcherry;* rol-6*(*su1006*)* | *unc-79(e1068) pat-2(st567)* |
| YW743 | tpEx300[*P_unc-54_pat-2::gfp;* rol-6*(*su1006*)]* | *unc-79(e1068) pat-2(st567)* |
| YW842 | tpEx315[*P_unc-54_moesin::gfp;* rol-6*(*su1006*)]* | wild-type |
| YW871 | tpEx312[*P_pat-2_pat-2Δcyto::gfp;* rol-6*(*su1006*)]* | wild-type |
| YW935 | tpEx307[*P_unc-54_ced-1::gfp;* rol-6*(*su1006*)]* | *ced-1(e1735)* |
| YW941 | tpEx314[*P_pat-2_pat-2Δcyto::gfp;* rol-6*(*su1006*)]* | *unc-79(e1068) pat-2(st567)* |
| YW974 | tpEx316[*P_unc-54_gfp::cdc-42;* rol-6*(*su1006*)]* | wild-type |
| YW985 | tpEx319[*P_pat-2_nls::gfp; P_unc-54_ced-1::mrfp;*  *r*ol-6*(*su1006*)]* | wild-type |
| YW1045 | tpEx283[*P_pat-2_pat-2::gfp;* rol-6*(*su1006*)]* | *ced-1(e1735); ced-5(n1812)* |
| YW1048 | tpEx334[*P_pat- 2_pat-2::mcherry; P_unc-54_gfp::cdc-42;* rol-6*(*su1006*)]* | *unc-79(e1068) pat-2(st567)* |
| YW1073 | tpEx285[*P_unc-54_pat-2::mcherry; P_ajm-1_pat-2::gfp; r*ol-6*(*su1006*)]* | wild-type |
| YW1108 | tpEx308[*P_ajm-1_ced-1::gfp;* rol-6*(*su1006*)]* | *ced-1(e1735)* |
| YW1133 | tpEx288[*P_pat-2_pat-2::gfp;* rol-6*(*su1006*)];*  tpEx333[*P_ajm-1_pat-2::gfp; P_sur-5_rfp]* | *unc-79(e1068) pat-2(st567)* |
| YW1135 | tpEx311[*P_hsp_pat-2(ex)::mcherry;* rol-6*(*su1006*)]* | wild-type |
| YW1178 | tpEx287[*P_pat-2_pat-2::gfp;* rol-6*(*su1006*)]* | wild-type |
| YW1183 | tpEx320[*P_hsp_pat-2(ex)::mcherry;* rol-6*(*su1006*)]* | *ced-1(e1735); ced-5(n1812)* |
| YW1733 | tpEx284[*P_unc-54_nls::gfp;* rol-6*(*su1006*)]* | *ced-1(e1735)* |
| YW1734 | tpEx282[*P_pat-2_nls::gfp; P_hsp_annexin V::mrfp*  *; r*ol-6*(*su1006*)]* | wild-type |
| YW1735 | tpEx286[*P_pat-2_pat-2::mcherry; P_unc-54_gfp::cdc-42; r*ol-6*(*su1006*)]* | wild-type |
| YW1738 | tpEx304[*P_unc-54_ina-1::gfp;* rol-6*(*su1006*)]* | *ina-1(gm144)* |
| YW1739 | tpEx305[*P_ajm-1_ina-1::gfp;* rol-6*(*su1006*)]* | *ina-1(gm144)* |
| YW1740 | tpEx306[*P_ced-1_ced-1::gfp;* rol-6*(*su1006*)]* | *ced-1(e1735)* |
| YW1741 | tpEx309[*P_ajm-1_pat-2::gfp;* rol-6*(*su1006*)]* | *ced-1(e1735)* |
| YW1742 | tpEx310[*P_hsp_gfp::cdc-42;* rol-6*(*su1006*)]* | *ced-1(e1735)* |
| YW1745 | tpEx321[*P_hsp_pat-2(ex)::mcherry; P_hsp_ ina-1(N)::gfp; r*ol-6*(*su1006*)]* | *ced-1(e1735); ced-5(n1812)* |
| YW1746 | tpEx288[*P_pat-2_pat-2::gfp;* rol-6*(*su1006*)];*  tpEx322[*P_unc-54_myri::mrfp;* rol-6*(*su1006*)]* | *unc-79(e1068) pat-2(st567)* |
| YW1747 | tpEx323[*P_unc-54_myri::mrfp;* rol-6*(*su1006*)]* | *cdc-42(gk388)/mIn1[mIs14 dpy-10(e128)]* |
| YW1748 | tpEx324[*P_unc-54_myri::mrfp; P_emb-9_gfp;* rol-6*(*su1006*)]* | *ced-1(e1735)* |
| YW1749 | tpEx325[*P_hsp_ pat-2(ex)::mcherry; P_ced-1_ced-1::gfp; r*ol-6*(*su1006*)]* | wild-type |
| YW1750 | tpEx327[*P_unc-54_gfp::cdc-42; r*ol-6*(*su1006*)]* | *cdc-42(gk388)/mIn1[mIs14 dpy-10(e128)]* |
| YW1751 | tpEx328[*P_ajm-1_gfp::cdc-42; r*ol-6*(*su1006*)]* | *cdc-42(gk388)/mIn1[mIs14 dpy-10(e128)]* |
| YW1752 | tpEx329[*P_hsp_ced-10V12;* pTG96[*sur-5::gfp*]*]* | *unc-79(e1068) pat-2(st567)/dpy-17(e164)* |
| YW1820 | tpEx343[*P_ajm-1_gfp::cdc-42; r*ol-6*(*su1006*)]* | *ina-1(gm144)* |
| YW1821 | tpEx344[P_hsp_gfp::cdc-42*; r*ol-6*(*su1006*)]* | wild-type |
| YW1822 | tpEx345[P_hsp_gfp::cdc-42*; r*ol-6*(*su1006*)]* | *ced-10(n3246)* |
| YW1823 | tpEx346[P_hsp_gfp::cdc-42*; r*ol-6*(*su1006*)]* | *ced-1(e1735)* |
| YW1824 | tpEx331[*P_hsp_ced-10V12;* P_sur-5_::rfp*]* | *ced-10(tm597)/dpy-13(e184)* |
