# Supplementary material for: Integrin α PAT-2/CDC-42 Signaling Is Required for Muscle-Mediated Clearance of Apoptotic Cells in Caenorhabditis elegans
Source: PLoS Genet. 2012 May 17;8(5):e1002663. doi: 10.1371/journal.pgen.1002663 (PMC3355063; doi:10.1371/journal.pgen.1002663)
Supplement: Text S1 — pat-2 does not appear to be expressed in embryonic apoptotic cells. (RTF) [file pgen.1002663.s013.rtf]

Supporting Information 
Text S1

pat-2 does not appear to be expressed in embryonic apoptotic cells
During apoptosis in C. elegans, dying cells show an “eat me” signal for engulfment before displaying the apoptotic morphology [1, 2]. We used the annexin V::mRFP fusion protein, which binds to externalized PS on the surface of apoptotic cells (D. Xue and X. Wang, personal communication), to label dying cells at the early apoptotic stage when their refractile apoptotic appearance has not yet become obvious under DIC optics. To determine whether pat-2 was expressed in apoptotic cells during embryogenesis, we co-expressed in wild-type embryos the transgenes Ppat-2nls::gfp and Phspannexin V::mrfp, which results in expression and secretion of the Annexin V::mRFP fusion protein under the control of the heat-shock promoter Phsp. ,The GFP signal was not detectable in apoptotic cells labeled with Annexin V::mRFP or those showing a refractile morphology under DIC optics (Figure S1A) or in the apoptotic MSpppaaa cell corpse labeled with annexin V::mRFP (Figure S1B) and showing a refractile morphology under DIC optics (Figure S1B). 

References
1. Robertson A, Thomson N (1982) Morphology of programmed cell death in the ventral nerve cord of Caenorhabditis elegans larvae. J Embryol Exp Morphol 67: 89-100.
2. Hsu TY, Wu YC (2010) Engulfment of apoptotic cells in C. elegans is mediated by integrin alpha/SRC signaling. Curr Biol 20: 477-486.
